# Enhancing Type 2 Diabetes Management Involving Healthcare Professionals in Primary Care

**DOI:** 10.3390/healthcare14162633

**Published:** 2026-08-20

**Authors:** Mengyao Li, Lijian Wang

**Affiliations:** School of Public Policy and Administration, Xi’an Jiaotong University, Xi’an 710049, China

**Keywords:** diabetes management, primary care, health system, healthcare intervention, health outcome, type 2 diabetes mellitus

## Abstract

**Highlights:**

**What are the main findings?**

- This study identifies five key intervention categories for managing type 2 diabetes: workforce transformation, service content, re-designing service delivery systems, information and communication technology (ICT), and multifaceted approaches. Notably, most interventions were implemented in high-income economies. The interventions examined primarily targeted clinical, behavioral, and psychosocial outcomes, with clinical outcomes being the most widely addressed.
- The proportion of positive findings varied across intervention categories and outcome domains. The multifaceted interventions reported relatively more favorable findings for clinical outcomes, while re-designing the service delivery system interventions reported more favorable findings for behavioral outcomes. ICT interventions frequently reported favorable findings related to blood glucose indicators.

**What are the implications of the main findings?**

- Policymakers should tailor interventions to specific needs, select appropriate delivery methods, and integrate technology effectively to improve diabetes management.
- Comprehensive, multilevel approaches spanning individual, organizational, and societal levels are needed to achieve effective long-term outcomes.

**Abstract:**

Background: Current evidence on diabetes care interventions is either fragmented or focused on specific interventions targeting healthcare professionals or patients. This study aims to categorize diabetes care interventions involving physicians or nurses and their impact on health outcomes for individuals with diabetes. Additionally, it aimed to determine the proportion of favorable findings across healthcare intervention categories. Methods: This scoping review was conducted in primary care settings and guided by a health system framework. We searched articles from inception to June 2022 in databases including CENTRAL, MEDLINE, Embase, PsycINFO, and CINAHL. The classification of healthcare interventions was guided by the Cochrane Effective Practice and Organization of Care taxonomy and health system framework. Results: Results were reported following the PRISMA Extension for Scoping Reviews. From the initial pool of 13,406 articles, 116 met the eligibility criteria and reported interventions conducted across 119 countries or regions, of which 94 were high-level economies. Five healthcare intervention categories were identified: transforming the workforce; service content; re-designing the service delivery system; information and communication technology; and multifaceted. Patient health outcomes were classified into 3 overarching categories with 11 subcategories: clinical outcomes, behavioral outcomes, and psychosocial outcomes. The proportion of statistically significant favorable findings differed across intervention categories and outcome domains. Multifaceted interventions showed relatively higher proportions of favorable findings for clinical outcomes, whereas re-designing the service delivery system interventions showed relatively higher proportions for behavioral outcomes. ICT interventions frequently reported favorable findings related to blood glucose indicators. Conclusions: This study provides a comprehensive overview of healthcare interventions and health outcomes for diabetes in primary care settings. It emphasizes the importance of tailored interventions, delivery methods, and technology integration for effective diabetes management, advocating for a comprehensive approach from individual to societal levels.

## 1. Introduction

Diabetes mellitus presents a significant global challenge, with a prevalence of 10.5% among individuals aged 20–79 in 2021. There were 537 million diabetes patients worldwide in 2021, which will increase to 783 million in 2045 [1]. Type 2 diabetes constitutes 96% of all diabetes cases globally [2]. Diabetes not only impacts the individual health status but also imposes substantial healthcare costs. On a personal level, diabetes is associated with high morbidity and mortality, with adults suffering a cardiovascular risk two to four times greater than those without diabetes [3]. During the COVID-19 pandemic, the mortality rate among patients with diabetes was 2.3 times higher than in those without the condition [4]. The burden of disease is inequitable, with diabetes-related mortality rates and disability-adjusted life years nearly double in low- and middle-income countries compared to high-income countries [5]. Economically, the health expenditure costs due to diabetes have escalated from 32 billion to 996 billion between 2007 and 2021 [4]. Given these challenging circumstances, the effective management of diabetes is essential to mitigate and curb its burden.

The equitable distribution of a high-performing health workforce, coupled with a high-quality health system, is essential for effective and equitable chronic disease management, including diabetes [6]. Reorganizing the health system is a requisite to promote health outcomes, financial protection, and people-centered care for individuals with diabetes [7]. There is a global consensus to transition the diabetes care service to primary healthcare, given its principle of first contact and continuity [2]. An adequate primary care workforce is essential for delivering high-quality healthcare and achieving better outcomes in chronic management [8]. For instance, an increase in one primary care provider per 1000 adults correlated with a reduction in fasting plasma glucose levels [9]. Physicians and nurses play a pivotal role in providing primary healthcare across various countries [10]. Their involvement in diabetes management within a restructured health system is crucial for improving patient health.

Diabetes care has shifted from hospitals to primary care since the last century [11]. Substantive interventions, delivery modalities, and various strategies have been piloted and scaled up in primary care settings to improve diabetes management. Some of these interventions focus on patient outcomes by examining strategies to improve health outcomes, regardless of the professionals delivering the intervention [12,13]. Some interventions, however, concentrate on healthcare professionals, attempting to modify their knowledge or behavior to improve clinical practice, without necessarily focusing on the health outcomes of patients [14,15]. To the best of our knowledge, few studies consider healthcare professionals’ service delivery and patient outcomes simultaneously. One most similar systematic review published in 2001 reported professional and organizational interventions based on diabetes patient outcomes, but it was conducted more than twenty years ago [16]. There are few reviews from a broader perspective, targeting organizations or health systems, and the effects of interventions within these reviews focused only on clinical outcomes for people with diabetes [17,18]. Alongside promoting the physical health gauged by clinical metrics, successful diabetes care should also prioritize the wellbeing of individuals with diabetes, including health behavior and psychosocial health [19]. Other similar reviews tend to be disconnected, focusing on one special type of healthcare professional [20], particular health outcomes of patients [21], a particular location [22], or unique interventions [23,24]. There is scarce and fragmented literature on interventions that involve professionals and concurrently impact patients’ outcomes. Moreover, the health outcomes of patients should be more comprehensive rather than limited to clinical outcomes.

This review aims to synthesize diabetes care interventions from a broader health system perspective, involving healthcare professionals with patient health outcomes simultaneously. The research question guiding this review is as follows: in primary care, what is known from the existing literature regarding interventions for physicians or nurses and the types of outcomes for patients with type 2 diabetes? The specific research objectives are as follows: (1) to identify the basic characteristics of the interventions; (2) to identify the specific types of interventions in which the primary care physicians or nurses are engaged within the health system context; (3) to identify the more comprehensive types of patient health outcomes with diabetes in clinical, behavioral, and psychosocial domains; (4) to explore the relationship between intervention types and health outcomes from narrative synthesis.

## 2. Materials and Methods

### 2.1. Search Strategy

Systematic searches were conducted across several bibliographic databases, CENTRAL, MEDLINE, Embase, PsycINFO, and CINAHL, spanning from the inception of these databases to 20 June 2022. The search strategy was guided by the Medical subject headings (MeSH) and keywords such as diabetes, primary care, and intervention. The search strategy was initially developed and tested in the MEDLINE database via PubMed and then translated into other bibliographic databases. The detailed search strategies are provided in Tables S1–S5 in the Supplementary Materials.

### 2.2. Selection Criteria and Study Selection

Given our study interests, eligible studies must concentrate on type 2 diabetes patients in primary care settings, with a focus on patient health outcomes and interventions involving physicians or nurses. The selection criteria were formulated using the population, concept, and context (PCC) framework. The population included patients with type 2 diabetes worldwide. The concept focused on interventions delivered by or involving physicians or nurses aimed at improving diabetes care, and the context comprised primary care settings across countries and regions.

The detailed inclusion criteria were as follows: (1) studies involving patients with type 2 diabetes; (2) interventions targeting physicians or nurses as active participants in diabetes care delivery; (3) studies reporting patient health outcomes; (4) study designs including randomized controlled trials, non-randomized controlled trials, or quasi-experiments. Exclusion criteria helped refine the selection process. Articles were excluded if they (1) were reviews, proposals, pilot studies, conference abstracts, editorials, commentaries, dissertations, theses, letters, descriptive or qualitative studies, incomplete studies, or cross-sectional studies; (2) were not published in English; (3) did not involve physicians or nurses; or (4) were conducted in hospitals or outside of primary care settings. Cross-sectional, descriptive, and qualitative studies were excluded because they were not suitable for assessing intervention-related changes in patient outcomes. Primary care settings referred to frontline healthcare facilities providing first-contact care, such as general practices, community health centers, and primary healthcare centers.

Study selection involved two stages: title and abstract screening, followed by full-text screening. To ensure the selection quality, these stages were independently conducted by two authors. Discrepancies in eligibility between the two authors were resolved with the involvement of a third author to confirm consensus. After completing both selection stages, the final set of studies was included.

### 2.3. Data Extraction

Once the articles were selected, two authors collaborated to extract relevant data into an Excel spreadsheet. The extracted data included the author’s name, publication year, location, setting of the intervention, study design, detailed description of the intervention, health outcomes, and results of intervention. The extracted data was checked, and a consensus was achieved to ensure quality. In instances of disagreement, a third author was consulted to deliberate and help finalize the consensus. The results of the intervention were categorized as either positive or non-positive. A positive result was defined as a statistically significant improvement in a patient outcome in the favorable direction, either between the intervention and comparison groups or between pre-intervention and post-intervention measurement, depending on the study design. Results were classified as non-positive if they were not statistically significant, showed no improvement, or indicated a deterioration in the outcome.

### 2.4. Data Synthesis and Analysis Framework

**Figure 1:**
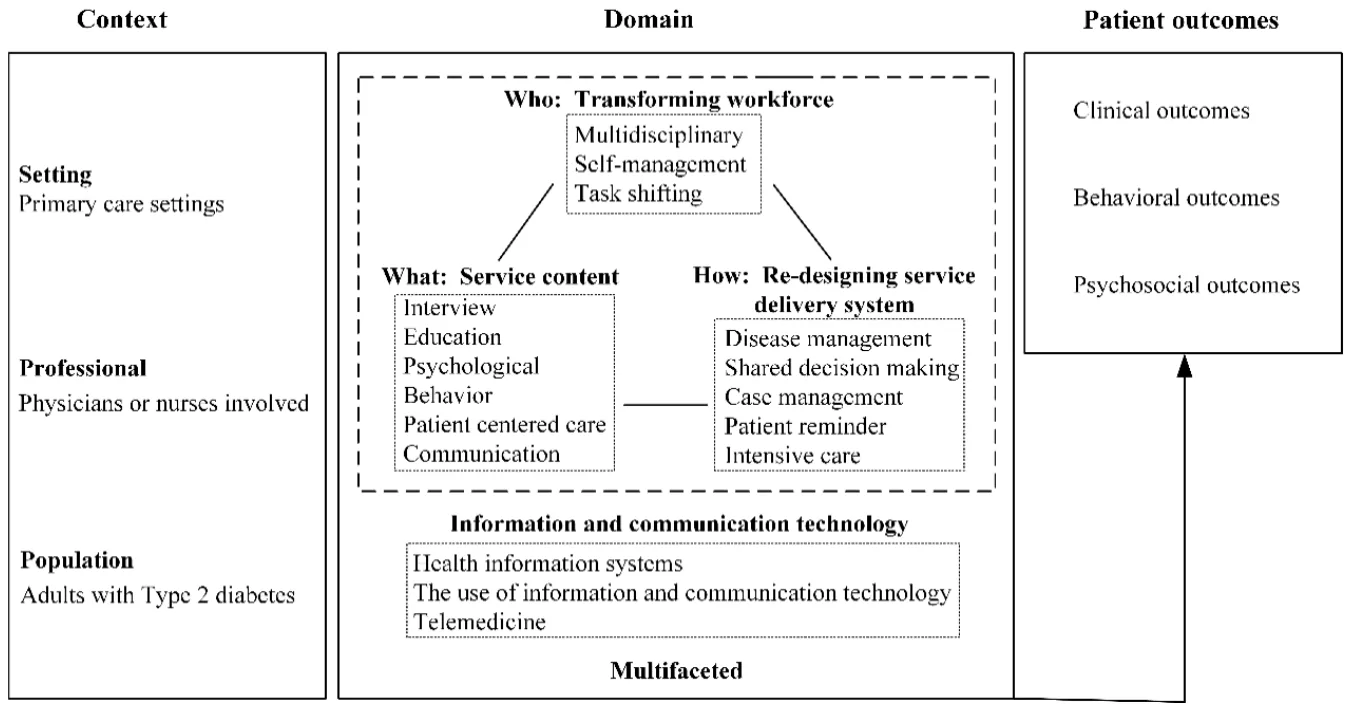
Framework of the data synthesis.

The review was reported according to PRISMA Extension for Scoping Reviews (PRISMA-ScR), a widely accepted reporting guideline for scoping reviews. After completion of the analyses, this review was retrospectively registered with the Open Science Framework (DOI: https://doi.org/10.17605/OSF.IO/82GCA). The framework for the data synthesis process is portrayed in Figure 1. First, we aggregated and summarized the location and publication years of the included articles. Second, we synthesized the intervention categories guided by the Cochrane Effective Practice and Organization of Care (EPOC) [25] under the framework of the health system performance assessment [26,27]. The EPOC review group is dedicated to enhancing professional practice and the delivery of effective healthcare services, aligning closely with the objectives of our study. The health-system-performance assessment framework facilitates the systematic categorization of interventions. The interventions were classified into five groups: transforming the workforce (who provides care); service content (what care is provided); re-designing the service delivery system (how care is delivered); information and communication technology; and multifaceted interventions. Following this, we synthesized the health outcomes for diabetes patients into three broad categories: clinical outcomes, behavioral outcomes, and psychosocial outcomes. Conclusively, we quantified the positive results for each health outcome within each intervention category.

## 3. Results

### 3.1. Characteristics of the Involved Studies

**Figure 2:**
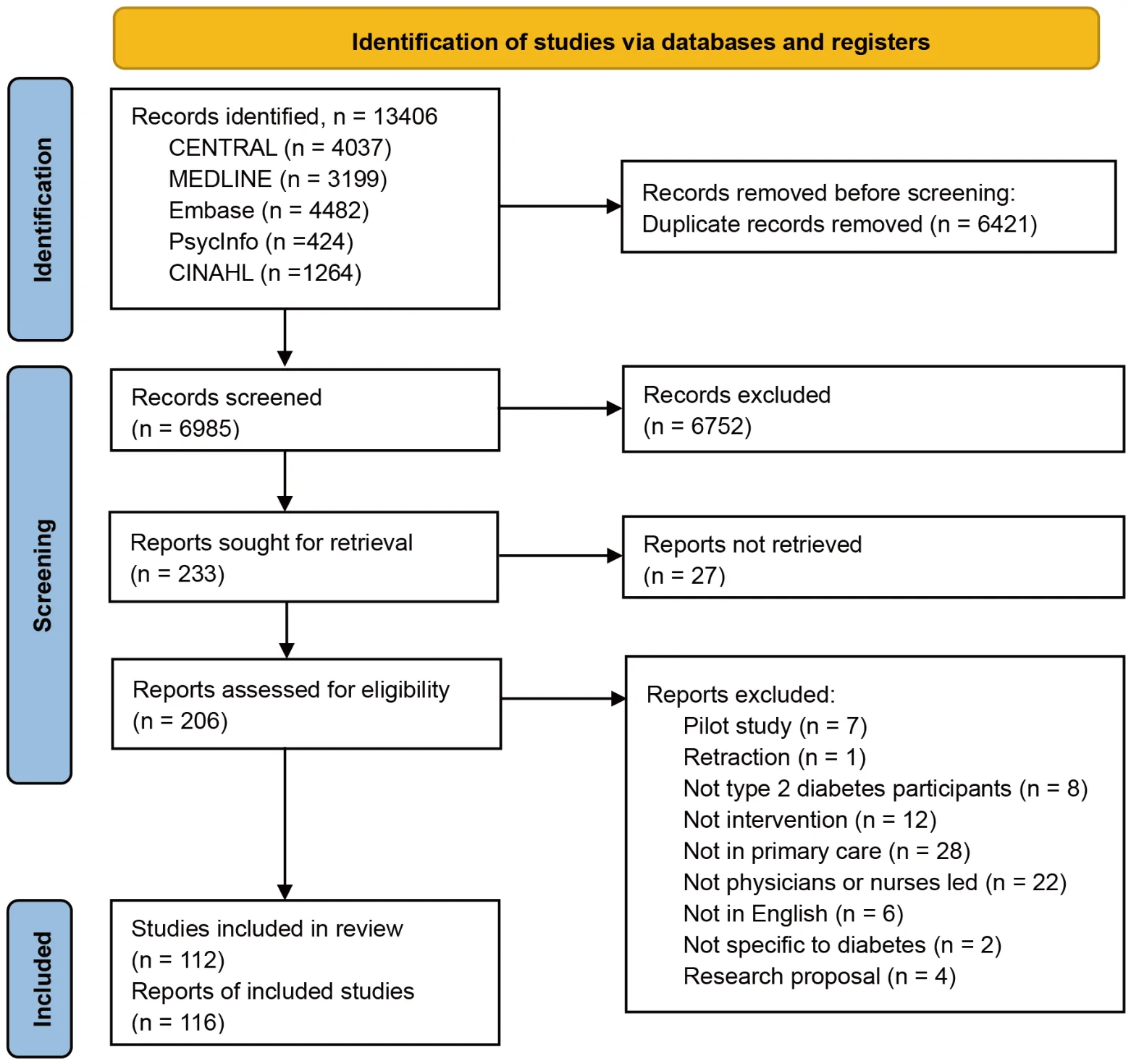
Review flow diagram.

The search strategy employed across five bibliographic databases yielded 13,406 articles. After the removal of duplicates and conducting title, abstract, and full-text screenings, a total of 116 articles encompassing 112 studies were selected for further synthesis and analysis. The process of review screening and eligibility flow diagram is portrayed in Figure 2.

**Figure 3:**
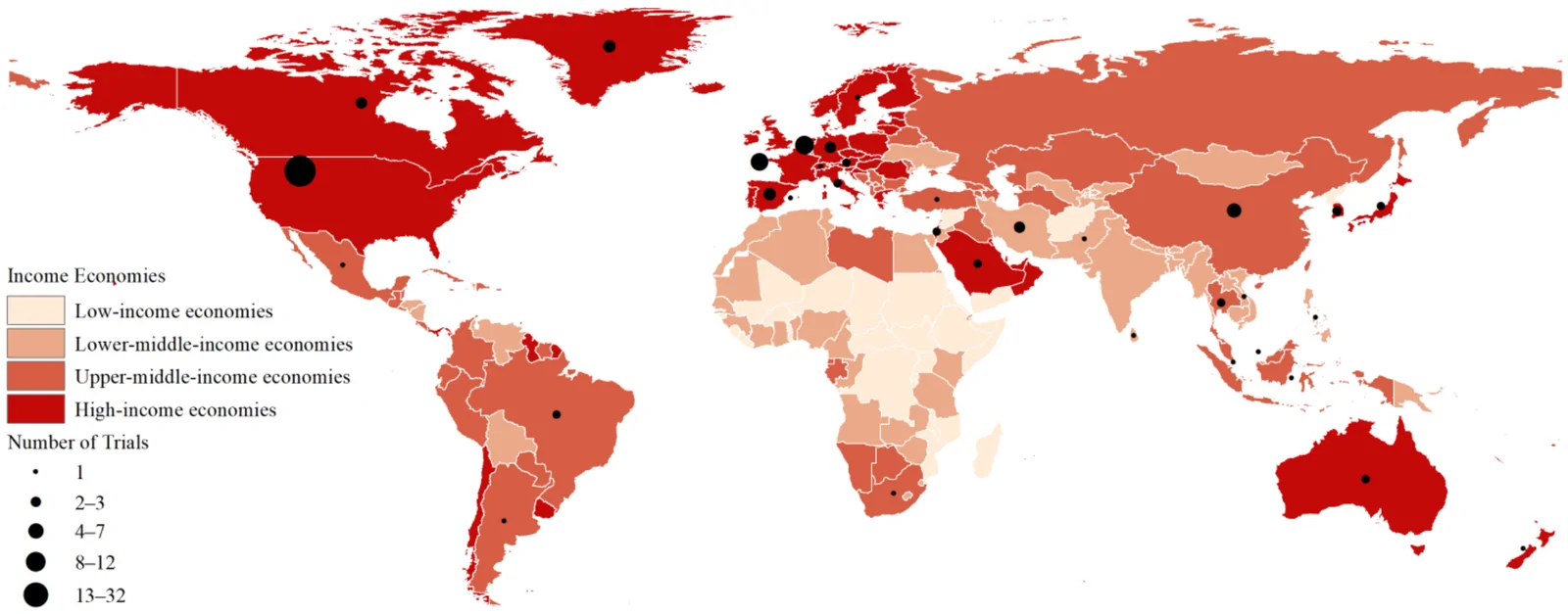
Number of published studies in diabetes care per country. The 112 included studies contributed 119 country-level entries because one study was conducted across eight countries, whereas each of the remaining 111 studies was conducted in a single country.

**Figure 4:**
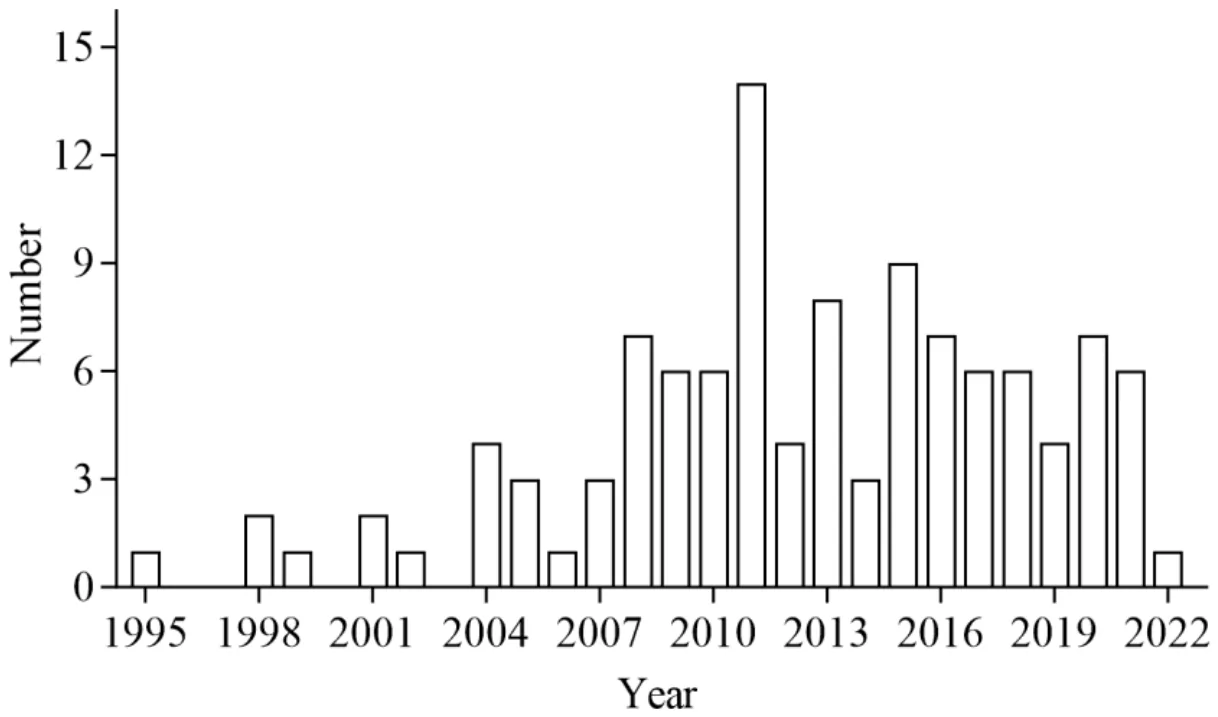
Number of published studies in diabetes care per year.

Among the 112 studies, the geographical distribution of where the interventions took place is detailed in Figure 3. These studies spanned 119 countries and regions, with Europe leading the count at 46, followed by North America with 36, Asia with 30, South America with 3, and Oceania with 3. Africa had the fewest studies, with only 1. In terms of economic status, 94 interventions were implemented in high-income economies, 21 in upper-middle-income economies, 4 in lower-middle-income economies, and none in low-income economies. The temporal distribution of these interventions, as depicted in Figure 4, shows that the first publication was in 1995. The number of interventions then gradually increased, peaking at 14 in 2011, and stabilized at around 6 per year until 2021.

### 3.2. Categories of Interventions

The interventions were categorized into five main groups, with the results summarized in Table 1. The categories and their respective intervention counts are as follows: who, transforming the workforce—25 interventions; what, service content—37 interventions; how, re-designing the service delivery system—17 interventions; information and communication technology—26 interventions; and multifaceted—7 interventions.

The transforming workforce category encompasses modifications in care provision and the allocation of health workers, featuring 17 multidisciplinary, 7 self-management strategies, and 1 task-shifting approach. The service content category elucidates the nature of services healthcare professionals deliver, including 10 interview techniques, 12 educational programs, 3 psychological interventions, 6 behavioral strategies, 3 patient-centered care approaches, and 3 combined methods involving 2 “education and behavior” and 1 “education and interview.” The re-designing the health service delivery system category highlights improvements in the care delivery process and the engagement between healthcare professionals and others, consisting of eight disease management programs, three shared decision-making strategies, three case management interventions, one patient reminder system, and two intensive care approaches. The information and communication technology category represents the digital tools healthcare organizations employ for healthcare management and delivery, comprising 3 health information systems, 15 applications of information and communication technology, and 8 telemedicine interventions. Lastly, the multifaceted category integrates various dimensions not specifically covered in the other categories.

### 3.3. Health Outcomes of Patients

Table 2 shows the health outcomes of patients reported in 116 articles. Notably, over 90% of these articles documented clinical outcomes, and almost 90% reported the blood glucose indicators, followed by non-laboratory measures (68.97%) and laboratory measures (63.79%). In terms of behavioral outcomes, 65.52% noted this measure. Within the behavioral outcomes category, process quality (31.90%) and health services utilization (30.17%) were the most prevalent outcome categories followed by treatment and medicine, and physical activity, at 26.72% and 16.38%, respectively. Psychosocial outcomes were covered in over half of the articles, with affective outcomes being the most frequently reported subcategory (30.17%). Satisfaction measures, cognitive–attitudinal outcomes, and quality of life were 24.14%, 12.07%, and 10.34%, respectively.

Regarding the positive outcomes across the main categories, the percentages of positive results reported for clinical outcomes, behavioral outcomes, and psychosocial outcomes were 65.14%, 46.05%, and 55.22%, respectively. Among the subcategories, the percentages of positive results varied significantly, ranging from 31.43% in health services utilization to 64.29% in cognitive–attitudinal outcomes.

### 3.4. Health Outcomes in Intervention Categories

**Figure 5:**
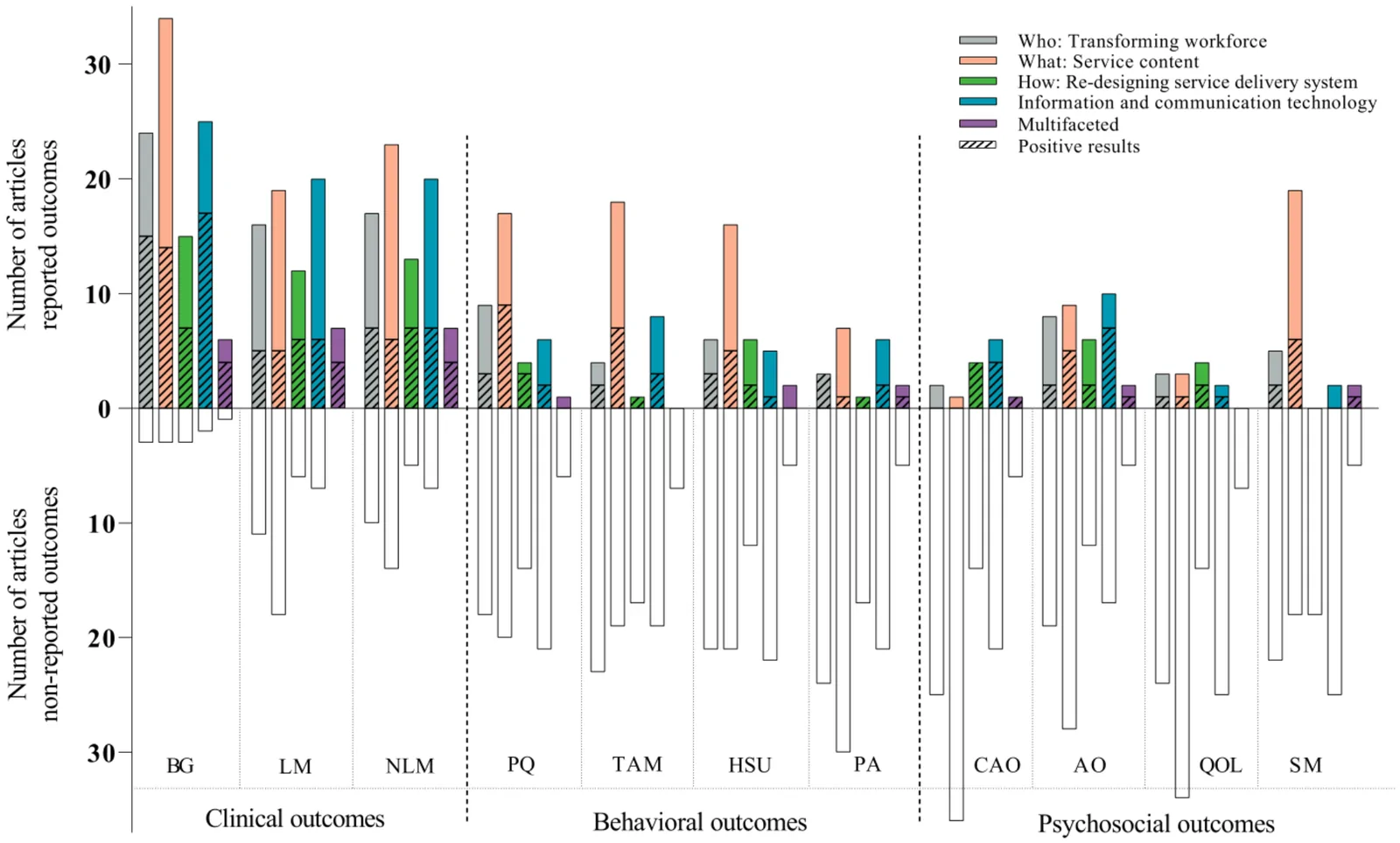
Health outcomes in each intervention category. BG: blood glucose; LM: laboratory measures; NLM: non-laboratory measures; PQ: process quality; TAM: treatment and medicine; HSU: health services utilization; PA: physical activity; CAO: cognitive–attitudinal outcomes; AO: affective outcomes; QOL: quality of life; SM: satisfaction measure.

**Figure 6:**
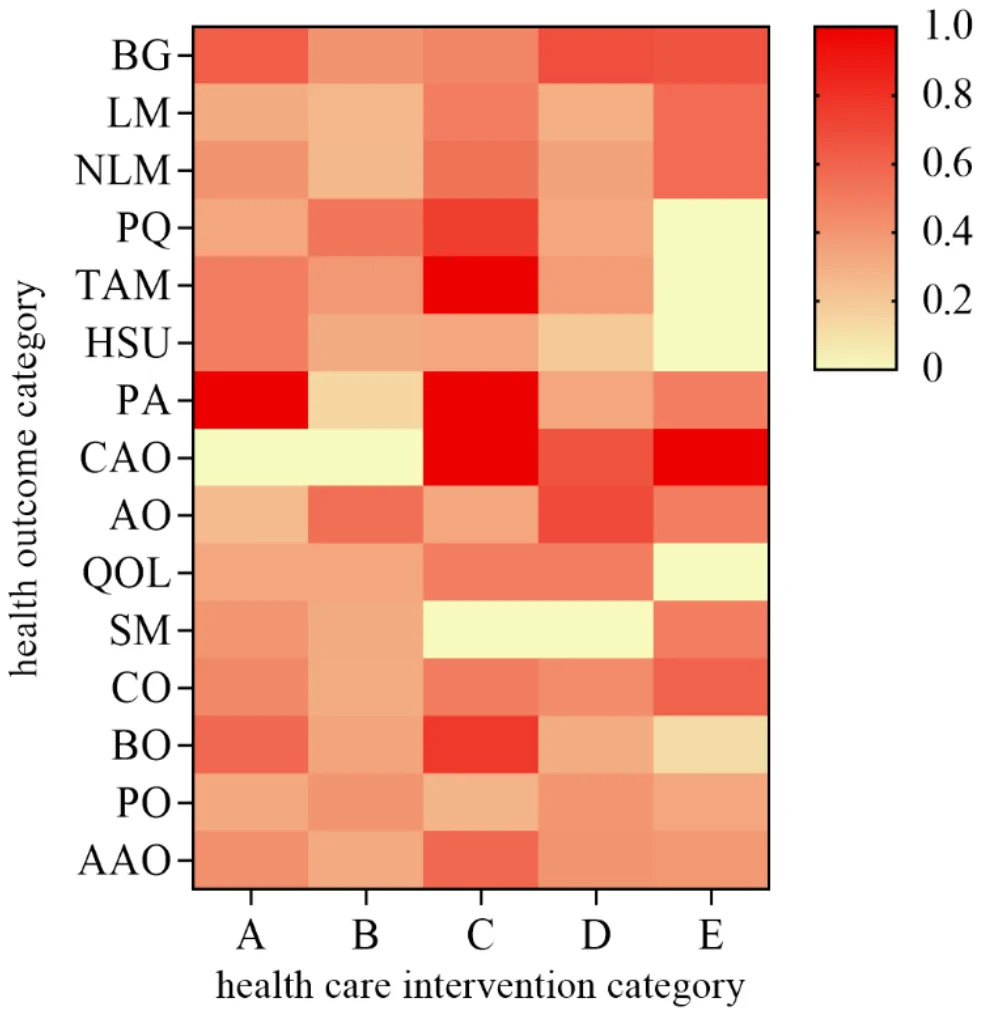
Positive percentage of each intervention category in health outcome categories. The positive percentage is the number of positive results as a percentage of the total number of results reported. BG: blood glucose; LM: laboratory measures; NLM: non-laboratory measures; PQ: process quality; TAM: treatment and medicine; HSU: health services utilization; PA: physical activity; CAO: cognitive–attitudinal outcomes; AO: affective outcomes; QOL: quality of life; SM: satisfaction measures; CO: clinical outcomes; BO: behavioral outcomes; PO: psychological outcomes; AAO: average of all outcomes; A: who, transforming workforce; B: what, service content; C: how, re-designing the service delivery system; D: information and communication technology; E: multifaceted.

Figure 5 portrays the health outcomes of patients across various intervention categories. Figure 6 shows the positive percentage of each intervention category in health outcomes, with darker colors indicating higher positive percentages. Across the five intervention categories, articles predominantly reported clinical outcomes. However, the five categories showed similar reporting of behavioral outcomes and psychosocial outcomes, with the exception of the service content category. The service content category notably reported higher instances of behavioral outcomes and the subcategory satisfaction measures within psychosocial outcomes.

Regarding the percentage of positive results, over 50% was achieved in 7 out of 11 health outcomes within both the re-designing the service delivery system and multifaceted categories. The re-designing the service delivery system category showed a relatively higher proportion of favorable findings across the 11 health outcome categories (58%). Notably, this category also reported a relatively higher proportion of favorable findings for behavioral outcomes. In clinical outcomes, the multifaceted category showed a higher proportion of favorable findings (60%). For psychosocial outcomes, the service content and the information and communication technology categories exhibited relatively higher positive percentages.

Within the subcategories of health outcomes, the information and communication technology category reported a relatively higher proportion of favorable findings for blood glucose indicators (68%). Similarly, this category also recorded a higher proportion of favorable findings for affective outcomes (70%). Regarding process quality, the re-designing the service delivery system category reported a higher proportion of favorable findings (75%). For satisfaction measures, the multifaceted category reported favorable findings in 50% of cases.

## 4. Discussion

This study performed a review to map out the diabetes care interventions involving physicians or nurses in primary care and to assess the health outcomes for diabetes patients over the past decades. A total of 116 articles representing 112 studies and covering 119 countries and regions were included. Of the 119 countries and regions represented, 94 (79.0%) were classified as high-income economies. We found that 32% of studies examined the intervention components of diabetes care services, specifically within the service content category. The proportion of positive findings varied across intervention categories and outcome domains. Multifaceted interventions were frequently associated with favorable clinical outcome findings, whereas re-designing the service delivery system interventions showed relatively more favorable findings for behavioral outcomes. Additionally, the category of information and communication technology reported favorable findings related to blood glucose indicators. Given the escalating global burden of diabetes [144], this review highlights the potential of health-system-based interventions by healthcare providers to enhance diabetes management in primary care.

The majority of diabetes care interventions were concentrated in high-income economies, despite the significant burden of diabetes and healthcare needs in low- and middle-income countries (LMICs). In line with the earlier review, the vast digital interventions for non-communicable diseases during the COVID-19 pandemic were predominantly conducted in high-income countries [145]. This geographic inequity was also illustrated by the fact that less than 10% of individuals in LMICs received guideline-based diabetes care [146]. Furthermore, diabetes-related mortality rates and disability-adjusted life-years were almost double in LMICs in 2019 [5]. Recent efforts have begun to address this geographic disparity, focusing not only on individual-level interventions but also on broader systemic changes, such as capacity building and improving the clinical practice environment [6,147]. Our findings underscore the urgent need to address geographic inequities, particularly the pressing burden in LMICs, and the importance of implementing effective and sustainable improvements in diabetes care.

In the interventions of diabetes care services, service content emerged as the most prevalent category, with the main interventions focusing on the nature of the services provided. Our results showed that interview and education were the two most frequent subcategories within service content. Motivational interviewing proved to have a positive impact on blood glucose control and diabetes-associated risk factors, such as weight loss [148,149]. Conversely, a meta-analysis revealed that patient education improved glycemic control only in a subgroup of patients whose baseline glycated hemoglobin (HbA1c) level was greater than 8% [150]. However, service content interventions showed relatively few positive findings across the reported health outcomes. The possible reason may be attributed to issues related to the process and fidelity of motivational interviewing [151], suggesting a need to identify the factors that effectively influence glycemic control. Nonetheless, a data-mining analysis suggested that the duration, content, and intensity of educational interventions did not significantly impact HbA1c [152]. Therefore, further diabetes care services should transcend the content itself and incorporate a comprehensive understanding of factors affecting efficacy and delivery methods.

The health outcomes for patients with diabetes exhibited considerable heterogeneity due to the varied measurement metrics employed. To mitigate this heterogeneity, the systematic review concentrated on a single intervention type and synthesized the corresponding health outcomes [149,153]. Alternatively, certain studies confined their analyses to specific health outcomes [154,155]. Similarly, the review encountered high heterogeneity, as heterogeneity signified the myriad of possibilities within healthcare [156]. To address this issue, our study organized the outcomes into three distinct groups: clinical, behavioral, and psychosocial. Since our scope was not restricted to interventions by physicians or nurses, we aimed to encompass a broader, more comprehensive array of health outcomes for diabetes patients. This inclusive approach facilitated a more nuanced understanding of potential health outcomes, thereby guiding the selection of targets for future diabetes care interventions.

Multifaceted interventions exhibited a relatively higher proportion of positive clinical outcomes. Our review included seven studies that employed a multifaceted, multifactorial, multicomponent, or multistrategic approach, all aimed at enhancing glucose control, improving diabetes care, or altering patient risk factors [137,138,139,140,141,142,143]. Multifaceted intervention was a prevalent service intervention in healthcare transformation, as evidenced by systematic reviews indicating that such interventions improved incontinence symptoms with low evidence quality [157] and improved diabetes care [158]. Despite their potential benefits, these interventions incur high costs due to increased workforce or resource demands. A cost-effectiveness analysis of multifaceted interventions in diabetes management revealed a cost-effectiveness ratio of €38,243, which exceeded the acceptable threshold of €20,000 [159]. In contrast, for patients with cardiovascular disease, the cost-effectiveness ratio was only 59% of the threshold [159]. Future multifaceted interventions in diabetes care must identify the specific patient groups that benefit most from these interventions to minimize unnecessary costs and ensure the sustained delivery of these interventions.

The re-designing the service delivery system demonstrated a relatively higher proportion of positive findings in behavioral outcomes and reported relatively favorable findings across health outcomes overall. This category, delineated by the EPOC [160], was benchmarked against care coordination and care process management and emphasized an interactive delivery modality. It included subcategories such as disease management, shared decision-making, etc. [95,96,97,99,100,103]. A meta-analysis showed that disease management in diabetes led to a reduction in HbA1c levels [161,162], alongside increased screening for retinopathy and foot complications [162]. A pivotal component of diabetes care management programs that contributed to their effectiveness was the increased frequency of patient contact [161], which aligns with the principles of patient-centered care. This notion is reinforced by the consensus report by the American Diabetes Association and the European Association for the Study of Diabetes, which prioritized patient-centered care in treatment delivery [163]. Furthermore, shared decision making has emerged as a strategy to foster patient-centered care, highlighting the exchange of information between providers and patients and centering on patients’ preferences and values [164]. In short, it is reasonable to conclude that enhancing patient engagement, emphasizing communication tailored to the patient’s context, and providing optional treatment choices are key facilitators of health outcomes for diabetes patients.

The category of information and communication technology demonstrated a relatively higher proportion of positive findings for blood glucose outcomes. Health information technology had the potential to improve healthcare service delivery and was recognized as a crucial building block for high-performing health systems [27,165]. Prior reviews had confirmed that information-technology-based diabetes management yielded positive clinical outcomes [166,167]. Additionally, a systematic review indicated that telemedicine in diabetes management could result in cost reductions and increased cost-effectiveness [168]. In the context of self-quarantine and social distancing situations, such as during the COVID-19 pandemic, information technology and social-media-based interventions had emerged as promising technologies to promote health and the sole effective method to deliver services during such crises [169,170]. With the proliferation of smartphones, medical device innovations, and enhancement of health information capacity, information-technology-based interventions are paramount for delivering diabetes care services in an uncertain environment while maintaining lower health costs.

Compared with the four functions of the health system [26], the interventions in this review predominantly fell within the domains of resource generation and service delivery, while governance and financing functions were not addressed in the studies included. It is a common phenomenon that interventions improving the quality of diabetes care often concentrated less on policy or environmental levels [171,172], which does not indicate that these levels are insignificant or neglected by society. In fact, diverse policies and strategies are being developed and implemented to address broader contextual concerns such as non-communicable diseases and to promote healthcare provider practices, all of which encompass diabetes management. Governments are instituting regulations to ensure the availability of essential medicines and to control medication prices, thereby making healthcare more accessible to the population [173]. Additionally, financial incentives, whether performance-based or not, are being employed to improve healthcare provider practices [174]. For instance, in Brazil, the decentralization of management and financing in primary healthcare has been associated with decreased diabetes-related mortality and hospitalizations in more developed regions [175]. Given the scope of this review, which is specifically tailored to synthesize interventions in diabetes management involving primary care physicians or nurses and their impact on patient health outcomes, it is evident that strategies at the policy or environmental level are notably underrepresented, despite their significance and close connection to the other functions within the health system.

This study provided a relatively comprehensive review of intervention categories and patients’ health outcomes with diabetes, as managed by physicians or nurses in primary settings. Our review synthesized evidence from randomized controlled trials, non-randomized controlled trials, and quasi-experimental studies, encompassing publications from their inception through June 2022. Nevertheless, this study does have certain limitations. Firstly, the inclusion criteria were limited to peer-reviewed publications in English, which may have excluded studies in other languages, potentially leading to incomplete information; nevertheless, similar to the findings of another systematic review, the impact of this exclusion is anticipated to be minimal [176]. Secondly, due to the nature of our review, which sought to encompass a variety of intervention types and study outcomes, a meta-analysis was deemed unsuitable for our heterogeneous dataset. Consequently, we employed a narrative synthesis to evaluate health outcomes and quantified the proportion of positive outcomes across each intervention category, thus providing partial insights into the research questions. Thirdly, the professionals involved in our study were limited to physicians or nurses, as they constitute the common health workforce whether in well-resourced places or not. Further research should consider incorporating pharmacists, educators, family members, peers, and other stakeholders to achieve a more holistic understanding of diabetes management within primary care contexts. Lastly, the findings should be interpreted as a mapping of evidence available up to June 2022 rather than a comprehensive representation of the current evidence base. Although the findings provide a broader overview of intervention types and reported outcomes, it may not capture important interventions published after the search date, particularly in rapidly evolving areas such as telemedicine, internet-based care, and large language-model-supported practice. In addition, the included studies did not consistently report economic outcomes or cost-effectiveness information, and this review did not evaluate the alignment of identified interventions with the latest diabetes-management guidelines. Future research should incorporate economic evaluations and guideline-based assessments to better inform the implementation of diabetes-management interventions.

## 5. Conclusions

In terms of diabetes care involving physicians or nurses in primary care settings, we identified five intervention categories, transforming the workforce, service content, re-designing the service delivery system, information and communication technology, and multifaceted, along with three categories of patient health outcomes: clinical outcomes, behavioral outcomes, and psychosocial outcomes. Despite our understanding of these diverse categories and their associated health outcomes, there is no single intervention that can universally improve diabetes care and patient outcomes in the long term. Our findings highlight the importance of a comprehensive approach that considers the service content (what care should be provided), the modality of service delivery (how to deliver services and the coordination of services), and the service object (patients with varying conditions), coupled with enhancing the capacity of health information technology to deliver patient-centered diabetes care with high-quality health outcomes. In the pursuit of advancing diabetes care management, we call for the collaboration between researchers and policymakers to formulate a promising and comprehensive strategy that spans from the individual level to the community and broader societal contexts.

## Figures and Tables

**Table 1:** Categories of physicians or nurses involved in interventions in 112 studies.

Intervention Category	*n*	Subcategory	*n*
Who: transforming workforce	25	Multidisciplinary [28,29,30,31,32,33,34,35,36,37,38,39,40,41,42,43,44,45]	17
		Self-management [46,47,48,49,50,51,52,53]	7
		Task shifting [54]	1
What: service content	37	Interview [55,56,57,58,59,60,61,62,63,64]	10
		Education [65,66,67,68,69,70,71,72,73,74,75,76]	12
		Psychological [77,78,79]	3
		Behavior [80,81,82,83,84,85]	6
		Patient-centered care [86,87,88]	3
		Combination ^1^ [89,90,91]	3
How: re-designing service delivery system	17	Disease management [92,93,94,95,96,97,98,99]	8
		Shared decision making [100,101,102,103]	3
		Case management [104,105,106]	3
		Patient reminder [107]	1
		Intensive care [108,109]	2
Information and communication technology	26	Health information systems [110,111,112]	3
		The use of information and communication technology [113,114,115,116,117,118,119,120,121,122,123,124,125,126,127,128]	15
		Telemedicine [129,130,131,132,133,134,135,136]	8
Multifaceted	7	[137,138,139,140,141,142,143]	

Note: ^1^ three combinations contain two “education and behavior” and one “education and interview”; details of the studies included in each category are provided in Supplementary Table S6.

**Table 2:** Distribution of reported health outcomes and positive findings across 116 articles.

Outcome Category/Subcategory	Articles Reporting the Outcome, *n* (% of 116 Articles)	Articles Reporting Positive Findings, *n* (% of Articles Reporting the Outcome)
Clinical outcomes	109 (93.97%)	71 (65.14%)
Blood glucose (BG)	104 (89.66%)	57 (54.81%)
Laboratory measures (LM)	74 (63.79%)	26 (35.14%)
Non-laboratory measures (NLM)	80 (68.97%)	31 (38.75%)
Behavioral outcomes	76 (65.52%)	35 (46.05%)
Process quality (PQ)	37 (31.90%)	17 (45.95%)
Treatment and medicine (TAM)	31 (26.72%)	13 (41.94%)
Health services utilization (HSU)	35 (30.17%)	11 (31.43%)
Physical activity (PA)	19 (16.38%)	8 (42.11%)
Psychosocial outcomes	67 (57.76%)	37 (55.22%)
Cognitive–attitudinal outcomes (CAO)	14 (12.07%)	9 (64.29%)
Affective outcomes (AO)	35 (30.17%)	17 (48.57%)
Quality of life (QOL)	12 (10.34%)	5 (41.67%)
Satisfaction measures (SM)	28 (24.14%)	9 (32.14%)

## Data Availability

No new data were created or analyzed in this study. Data sharing is not applicable to this article.

## Supplementary Materials

The following supporting information can be downloaded at: https://www.mdpi.com/article/10.3390/healthcare14162633/s1, Table S1: Search strategy in CENTRAL; Table S2: Search strategy in MEDLINE; Table S3: Search strategy in Embase; Table S4: Search strategy in PsycINFO; Table S5: Search strategy in CINAHL; Table S6: Basic characteristics of the interventions in the 112 included studies.

## References

[1] Sun H., Saeedi P., Karuranga S., Pinkepank M., Ogurtsova K., Duncan B.B., Stein C., Basit A., Chan J.C.N., Mbanya J.C. (2022). IDF Diabetes Atlas: Global, regional and country-level diabetes prevalence estimates for 2021 and projections for 2045. Diabetes Res. Clin. Pract..

[2] Varghese C., Nongkynrih B., Onakpoya I., McCall M., Barkley S., Collins T.E. (2019). Better health and wellbeing for billion more people: Integrating non-communicable diseases in primary care. BMJ.

[3] Dal Canto E., Ceriello A., Rydén L., Ferrini M., Hansen T.B., Schnell O., Standl E., Beulens J.W. (2019). Diabetes as a cardiovascular risk factor: An overview of global trends of macro and micro vascular complications. Eur. J. Prev. Cardiol..

[4] International Diabetes Federation (2021). IDF Diabetes Atlas.

[5] GBD Diseases and Injuries Collaborators (2020). Global burden of 369 diseases and injuries in 204 countries and territories, 1990–2019: A systematic analysis for the Global Burden of Disease Study 2019. Lancet.

[6] Walker A.F., Graham S., Maple-Brown L., Egede L.E., Campbell J.A., Walker R.J., Wade A.N., Mbanya J.C., Long J.A., Yajnik C. (2023). Interventions to address global inequity in diabetes: International progress. Lancet.

[7] Samb B., Desai N., Nishtar S., Mendis S., Bekedam H., Wright A., Hsu J., Martiniuk A., Celletti F., Patel K. (2010). Prevention and management of chronic disease: A litmus test for health-systems strengthening in low-income and middle-income countries. Lancet.

[8] Harris P., Kirkland R., Masanja S., Le Feuvre P., Montgomery S., Ansbro É., Woodman M., Harris M. (2022). Strengthening the primary care workforce to deliver high-quality care for non-communicable diseases in refugee settings: Lessons learnt from a UNHCR partnership. BMJ Glob. Health.

[9] Farzadfar F., Murray C.J.L., Gakidou E., Bossert T., Namdaritabar H., Alikhani S., Moradi G., Delavari A., Jamshidi H., Ezzati M. (2012). Effectiveness of diabetes and hypertension management by rural primary health-care workers (Behvarz workers) in Iran: A nationally representative observational study. Lancet.

[10] Freund T., Everett C., Griffiths P., Hudon C., Naccarella L., Laurant M. (2015). Skill mix, roles and remuneration in the primary care workforce: Who are the healthcare professionals in the primary care teams across the world?. Int. J. Nurs. Stud..

[11] Goyder E.C., McNally P.G., Drucquer M., Spiers N., Botha J.L. (1998). Shifting of care for diabetes from secondary to primary care, 1990–1995: Review of general practices. BMJ.

[12] Haider R., Sudini L., Chow C.K., Cheung N.W. (2019). Mobile phone text messaging in improving glycaemic control for patients with type 2 diabetes mellitus: A systematic review and meta-analysis. Diabetes Res. Clin. Pract..

[13] Eberle C., Stichling S. (2021). Clinical Improvements by Telemedicine Interventions Managing Type 1 and Type 2 Diabetes: Systematic Meta-review. J. Med. Internet Res..

[14] Crang M.C., Savage T.A., Schraeder C. (2022). An Educational Intervention for Mental Health Staff to Assess Clients’ Diabetic Self-Care Skills for Self-Management and Safe Transition into the Community. J. Psychosoc. Nurs. Ment. Health Serv..

[15] Daud M.H., Ramli A.S., Abdul-Razak S., Haniff J., Sidik T., Hatta N., Mahmood S., Lakshmanan S. (2020). Effectiveness of the empower-par intervention on primary care providers’ adherence to clinical practice guideline on the management of type 2 diabetes mellitus: A pragmatic cluster randomised controlled trial. Open Access Maced. J. Med. Sci..

[16] Renders C.M., Valk G.D., Griffin S.J., Wagner E.H., Eijk Van J.T., Assendelft W.J.J. (2001). Interventions to Improve the Management of Diabetes in Primary Care, Outpatient, and Community Settings. Diabetes Care.

[17] Tricco A.C., Ivers N.M., Grimshaw J.M., Moher D., Turner L., Galipeau J., Halperin I., Vachon B., Ramsay T., Manns B. (2012). Effectiveness of quality improvement strategies on the management of diabetes: A systematic review and meta-analysis. Lancet.

[18] Konnyu K.J., Yogasingam S., Lépine J., Sullivan K., Alabousi M., Edwards A., Hillmer M., Karunananthan S., Lavis J.N., Linklater S. (2023). Quality improvement strategies for diabetes care: Effects on outcomes for adults living with diabetes. Cochrane Database Syst. Rev..

[19] American Diabetes Association Professional Practice Committee for Diabetes (2023). 5. Facilitating Positive Health Behaviors and Well-being to Improve Health Outcomes: Standards of Care in Diabetes—2024. Diabetes Care.

[20] Little T.V., Wang M.L., Castro E.M., Jiménez J., Rosal M.C. (2014). Community Health Worker Interventions for Latinos with Type 2 Diabetes: A Systematic Review of Randomized Controlled Trials. Curr. Diabetes Rep..

[21] Smalls B.L., Walker R.J., Bonilha H.S., Campbell J.A., Egede L.E. (2015). Community Interventions to Improve Glycemic Control in African Americans with Type 2 Diabetes: A Systemic Review. Glob. J. Health Sci..

[22] Faruqi N., Thomas L., Parker S., Harris-Roxas B., Taggart J., Spooner C., Wong V., Harris M. (2019). Primary health care provider–focused interventions for improving outcomes for people with type 2 diabetes: A rapid review. Public Health Res. Pract..

[23] Thepwongsa I., Kirby C., Schattner P., Shaw J., Piterman L. (2014). Type 2 diabetes continuing medical education for general practitioners: What works? A systematic review. Diabet. Med..

[24] Werfalli M., Raubenheimer P.J., Engel M., Musekiwa A., Bobrow K., Peer N., Hoegfeldt C., Kalula S., Kengne A.P., Levitt N.S. (2020). The effectiveness of peer and community health worker-led self-management support programs for improving diabetes health-related outcomes in adults in low- and-middle-income countries: A systematic review. Syst. Rev..

[25] Cochrane Effective Practice Organisation of Care EPOC Resources for Review Authors. https://epoc.cochrane.org/resources/epoc-resources-review-authors.

[26] Papanicolas I., Rajan D., Karanikolos M., Soucat A., Figueras J. (2022). Health System Performance Assessment: A Framework for Policy Analysis.

[27] Gilson L. (2012). Health Policy and Systems Research: A Methodology Reader.

[28] Al Asmary S.M., Al-Harbi T., Tourkmani A.M., Al Khashan H.I., Al-Qahtani H., Mishriky A., Bakhiet A., Al Nowaiser N.A. (2013). Impact of integrated care program on glycemic control and cardiovascular risk in adult patients with type 2 diabetes. J. Clin. Outcomes Manag..

[29] Berry D.C., Williams W., Hall E.G., Heroux R., Bennett-Lewis T. (2016). Imbedding Interdisciplinary Diabetes Group Visits Into a Community-Based Medical Setting. Diabetes Educ..

[30] Bray P., Cummings D.M., Morrissey S., Thompson D., Holbert D., Wilson K., Lukosius E., Tanenberg R. (2013). Improved outcomes in diabetes care for rural African Americans. Ann. Fam. Med..

[31] DePue J.D., Dunsiger S., Seiden A.D., Blume J., Rosen R.K., Goldstein M.G., Nu’usolia O., Tuitele J., McGarvey S.T. (2013). Nurse-community health worker team improves diabetes care in American Samoa: Results of a randomized controlled trial. Diabetes Care.

[32] du Pon E., El Azzati S., van Dooren A., Kleefstra N., Heerdink E., van Dulmen S. (2019). Effects of a Proactive Interdisciplinary Self-Management (PRISMA) program on medication adherence in patients with type 2 diabetes in primary care: A randomized controlled trial. Patient Prefer. Adherence.

[33] Edelman D., Fredrickson S.K., Melnyk S.D., Coffman C.J., Jeffreys A.S., Datta S., Jackson G.L., Harris A.C., Hamilton N.S., Stewart H. (2010). Medical clinics versus usual care for patients with both diabetes and hypertension: A randomized trial. Ann. Intern. Med..

[34] Groeneveld Y., Petri H., Hermans J., Springer M. (2001). An assessment of structured care assistance in the management of patients with type 2 diabetes in general practice. Scand. J. Prim. Health Care.

[35] Hiss R.G., Armbruster B.A., Gillard M.L., McClure L.A. (2007). Nurse care manager collaboration with community-based physicians providing diabetes care: A randomized controlled trial. Diabetes Educ..

[36] Maddigan S.L., Majumdar S.R., Guirguis L.M., Lewanczuk R.Z., Lee T.K., Toth E.L., Johnson J.A. (2004). Improvements in patient-reported outcomes associated with an intervention to enhance quality of care for rural patients with type 2 diabetes: Results of a controlled trial. Diabetes Care.

[37] Maislos M., Weisman D. (2004). Multidisciplinary approach to patients with poorly controlled type 2 diabetes mellitus: A prospective, randomized study. Acta Diabetol..

[38] Majumdar S.R., Guirguis L.M., Toth E.L., Lewanczuk R.Z., Lee T.K., Johnson J.A. (2003). Controlled trial of a multifaceted intervention for improving quality of care for rural patients with type 2 diabetes. Diabetes Care.

[39] Markle-Reid M., Ploeg J., Fraser K.D., Fisher K.A., Bartholomew A., Griffith L.E., Miklavcic J., Gafni A., Thabane L., Upshur R. (2018). Community Program Improves Quality of Life and Self-Management in Older Adults with Diabetes Mellitus and Comorbidity. J. Am. Geriatr. Soc..

[40] Moinfar Z., Sedaghat M., Abolhassani F., Sharifi V., Moinfar Z., Eftekhari S., Mirzaaghaee F. (2016). A collaborative care program for management of common mental disorders among diabetic patients in a primary healthcare setting. J. Public Health.

[41] Najar A.V., Tehrani H., Imamian H., Hakak H.M., Vahedian-Shahroodi M. (2017). Impact of establishing a communication network of family physicians on level of Hba1cand FBS in patients with diabetes: A randomized clinical trial. Iran. Red. Crescent Med. J..

[42] Ni Y., Liu S., Li J., Dong T., Tao L., Yuan L., Yang M. (2019). The Effects of Nurse-Led Multidisciplinary Team Management on Glycosylated Hemoglobin, Quality of Life, Hospitalization, and Help-Seeking Behavior of People with Diabetes Mellitus. J. Diabetes Res..

[43] Talavera G.A., Castañeda S.F., Mendoza P.M., Lopez-Gurrola M., Roesch S., Pichardo M.S., Garcia M.L., Muñoz F., Gallo L.C. (2021). Latinos understanding the need for adherence in diabetes (LUNA-D): A randomized controlled trial of an integrated team-based care intervention among Latinos with diabetes. Transl. Behav. Med..

[44] Taylor K.I., Oberle K.M., Crutcher R.A., Norton P.G. (2005). Promoting health in type 2 diabetes: Nurse-physician collaboration in primary care. Biol. Res. Nurs..

[45] Tourkmani A.M., Abdelhay O., Alkhashan H.I., Alaboud A.F., Bakhit A., Elsaid T., Alawad A., Alobaikan A., Alqahtani H., Alqahtani A. (2018). Impact of an integrated care program on glycemic control and cardiovascular risk factors in patients with type 2 diabetes in Saudi Arabia: An interventional parallel-group controlled study. BMC Fam. Pr..

[46] Brown S.A., Garcia A.A., Kouzekanani K., Hanis C.L. (2002). Culturally competent diabetes self-management education for Mexican Americans: The Starr County border health initiative. Diabetes Care.

[47] Fisher L., Polonsky W., Parkin C.G., Jelsovsky Z., Amstutz L., Wagner R.S. (2011). The impact of blood glucose monitoring on depression and distress in insulin-naïve patients with type 2 diabetes. Curr. Med. Res. Opin..

[48] Jayasuriya R., Pinidiyapathirage M.J., Jayawardena R., Kasturiratne A., de Zoysa P., Godamunne P., Gamage S., Wickremasinghe A.R. (2015). Translational research for Diabetes Self-Management in Sri Lanka: A randomized controlled trial. Prim. Care Diabetes.

[49] Jutterström L., Hörnsten Å., Sandström H., Stenlund H., Isaksson U. (2016). Nurse-led patient-centered self-management support improves HbA1c in patients with type 2 diabetes—A randomized study. Patient Educ. Couns..

[50] Olry de Labry Lima A., Bermúdez Tamayo C., Pastor Moreno G., Bolívar Muñoz J., Ruiz Pérez I., Johri M., Quesada Jiménez F., Cruz Vela P., de Los Ríos Álvarez A.M., Prados Quel M. (2017). Effectiveness of an intervention to improve diabetes self-management on clinical outcomes in patients with low educational level. Gac. Sanit..

[51] Polonsky W.H., Fisher L., Schikman C.H., Hinnen D.A., Parkin C.G., Jelsovsky Z., Axel-Schweitzer M., Petersen B., Wagner R.S. (2011). A structured self-monitoring of blood glucose approach in type 2 diabetes encourages more frequent, intensive, and effective physician interventions: Results from the STeP study. Diabetes Technol. Ther..

[52] Polonsky W.H., Fisher L., Schikman C.H., Hinnen D.A., Parkin C.G., Jelsovsky Z., Petersen B., Schweitzer M., Wagner R.S. (2011). Structured self-monitoring of blood glucose significantly reduces A1C levels in poorly controlled, noninsulin-treated type 2 diabetes: Results from the Structured Testing Program study. Diabetes Care.

[53] van Dijk-de Vries A., van Bokhoven M.A., Winkens B., Terluin B., Knottnerus J.A., van der Weijden T., van Eijk J.T. (2015). Lessons learnt from a cluster-randomised trial evaluating the effectiveness of Self-Management Support (SMS) delivered by practice nurses in routine diabetes care. BMJ Open.

[54] Houweling S.T., Kleefstra N., van Hateren K.J., Groenier K.H., Meyboom-de Jong B., Bilo H.J. (2011). Can diabetes management be safely transferred to practice nurses in a primary care setting? A randomised controlled trial. J. Clin. Nurs..

[55] Akturan S., Kaya Ç., Ünalan P.C., Akman M. (2017). The effect of the BATHE interview technique on the empowerment of diabetic patients in primary care: A cluster randomised controlled study. Prim. Care Diabetes.

[56] De Greef K., Deforche B., Tudor-Locke C., De Bourdeaudhuij I. (2011). Increasing physical activity in Belgian type 2 diabetes patients: A three-arm randomized controlled trial. Int. J. Behav. Med..

[57] Farmer A., Hardeman W., Hughes D., Prevost A.T., Kim Y., Craven A., Oke J., Boase S., Selwood M., Kellar I. (2012). An explanatory randomised controlled trial of a nurse-led, consultation-based intervention to support patients with adherence to taking glucose lowering medication for type 2 diabetes. BMC Fam. Pract..

[58] Gabbay R.A., Añel-Tiangco R.M., Dellasega C., Mauger D.T., Adelman A., Van Horn D.H. (2013). Diabetes nurse case management and motivational interviewing for change (DYNAMIC): Results of a 2-year randomized controlled pragmatic trial. J. Diabetes.

[59] Jansink R., Braspenning J., Keizer E., van der Weijden T., Elwyn G., Grol R. (2013). No identifiable Hb1Ac or lifestyle change after a comprehensive diabetes programme including motivational interviewing: A cluster randomised trial. Scand. J. Prim. Health Care.

[60] Juul L., Maindal H.T., Zoffmann V., Frydenberg M., Sandbaek A. (2014). Effectiveness of a training course for general practice nurses in motivation support in type 2 diabetes care: A cluster-randomised trial. PLoS ONE.

[61] Lohmann H., Siersma V., Olivarius N.F. (2010). Fitness consultations in routine care of patients with type 2 diabetes in general practice: An 18-month non-randomised intervention study. BMC Fam. Pr..

[62] Mons U., Raum E., Krämer H.U., Rüter G., Rothenbacher D., Rosemann T., Szecsenyi J., Brenner H. (2013). Effectiveness of a supportive telephone counseling intervention in type 2 diabetes patients: Randomized controlled study. PLoS ONE.

[63] Rubak S., Sandbaek A., Lauritzen T., Borch-Johnsen K., Christensen B. (2009). General practitioners trained in motivational interviewing can positively affect the attitude to behaviour change in people with type 2 diabetes. One year follow-up of an RCT, ADDITION Denmark. Scand. J. Prim. Health Care.

[64] Young H.M., Miyamoto S., Dharmar M., Tang-Feldman Y. (2020). Nurse Coaching and Mobile Health Compared With Usual Care to Improve Diabetes Self-Efficacy for Persons With Type 2 Diabetes: Randomized Controlled Trial. JMIR Mhealth Uhealth.

[65] Blackberry I.D., Furler J.S., Best J.D., Chondros P., Vale M., Walker C., Dunning T., Segal L., Dunbar J., Audehm R. (2013). Effectiveness of general practice based, practice nurse led telephone coaching on glycaemic control of type 2 diabetes: The Patient Engagement and Coaching for Health (PEACH) pragmatic cluster randomised controlled trial. BMJ.

[66] Cabré Font C., Colungo Francia C., Vinagre Torres I., Jansà i Morató M., Conget Donlo I. (2021). A Therapeutic Education Program with a Diabetes Specialist Nurse for Type 2 Diabetes Patients Using Insulin in a Primary Care Setting. A Diabetes Education Program with a Diabetes Specialist Nurse in a Primary Care Setting. Endocrinol. Diabetes Nutr. (Engl. Ed.).

[67] De la Fuente Coria M.C., Cruz-Cobo C., Santi-Cano M.J. (2020). Effectiveness of a primary care nurse delivered educational intervention for patients with type 2 diabetes mellitus in promoting metabolic control and compliance with long-term therapeutic targets: Randomised controlled trial. Int. J. Nurs. Stud..

[68] Grillo Mde F., Neumann C.R., Scain S.F., Rozeno R.F., Beloli L., Perinetto T., Gross J.L., Leitão C.B. (2016). Diabetes education in primary care: A randomized clinical trial. Cad. Saude Publica.

[69] Martos-Cabrera M.B., Gómez-Urquiza J.L., Cañadas-González G., Romero-Bejar J.L., Suleiman-Martos N., Cañadas-De la Fuente G.A., Albendín-García L. (2021). Nursing-Intense Health Education Intervention for Persons with Type 2 Diabetes: A Quasi-Experimental Study. Healthcare.

[70] Moreo K., Sapir T., Greene L. (2015). Applying Quality Improvement into Systems-based Learning to Improve Diabetes Outcomes in Primary Care. BMJ Qual. Improv. Rep..

[71] Odnoletkova I., Goderis G., Nobels F., Fieuws S., Aertgeerts B., Annemans L., Ramaekers D. (2016). Optimizing diabetes control in people with Type 2 diabetes through nurse-led telecoaching. Diabet. Med..

[72] Reutens A.T., Hutchinson R., Van Binh T., Cockram C., Deerochanawong C., Ho L.T., Ji L., Khalid B.A., Kong A.P., Lim-Abrahan M.A. (2012). The GIANT study, a cluster-randomised controlled trial of efficacy of education of doctors about type 2 diabetes mellitus management guidelines in primary care practice. Diabetes Res. Clin. Pr..

[73] Sturt J.A., Whitlock S., Fox C., Hearnshaw H., Farmer A.J., Wakelin M., Eldridge S., Griffiths F., Dale J. (2008). Effects of the Diabetes Manual 1:1 structured education in primary care. Diabet. Med..

[74] Weinberger M., Kirkman M.S., Samsa G.P., Shortliffe E.A., Landsman P.B., Cowper P.A., Simel D.L., Feussner J.R. (1995). A nurse-coordinated intervention for primary care patients with non-insulin-dependent diabetes mellitus: Impact on glycemic control and health-related quality of life. J. Gen. Intern. Med..

[75] Woodard L., Amspoker A.B., Hundt N.E., Gordon H.S., Hertz B., Odom E., Utech A., Razjouyan J., Rajan S.S., Kamdar N. (2022). Comparison of Collaborative Goal Setting With Enhanced Education for Managing Diabetes-Associated Distress and Hemoglobin A1c Levels: A Randomized Clinical Trial. JAMA Netw. Open.

[76] Zandiyeh Z., Hedayati B., Zare E. (2015). Effect of public health nurses’ educational intervention on self-care of the patients with type 2 diabetes. J. Educ. Health Promot..

[77] Chiu C.J., Hu Y.H., Wray L.A., Beverly E.A., Yang Y.C., Wu J.S., Lu F.H. (2016). Dissemination of evidence-base minimal psychological intervention for diabetes management in Taiwan adults with type 2 diabetes. Int. J. Clin. Exp. Med..

[78] Ismail K., Winkley K., de Zoysa N., Patel A., Heslin M., Graves H., Thomas S., Stringer D., Stahl D., Amiel S.A. (2018). Nurse-led psychological intervention for type 2 diabetes: A cluster randomised controlled trial (Diabetes-6 study) in primary care. Br. J. Gen. Pr..

[79] Lamers F., Jonkers C.C., Bosma H., Knottnerus J.A., van Eijk J.T. (2011). Treating depression in diabetes patients: Does a nurse-administered minimal psychological intervention affect diabetes-specific quality of life and glycaemic control? A randomized controlled trial. J. Adv. Nurs..

[80] Edelman D., Dolor R.J., Coffman C.J., Pereira K.C., Granger B.B., Lindquist J.H., Neary A.M., Harris A.J., Bosworth H.B. (2015). Nurse-led behavioral management of diabetes and hypertension in community practices: A randomized trial. J. Gen. Intern. Med..

[81] Frosch D.L., Uy V., Ochoa S., Mangione C.M. (2011). Evaluation of a behavior support intervention for patients with poorly controlled diabetes. Arch. Intern. Med..

[82] Kim M.T., Kim K.B., Huh B., Nguyen T., Han H.-R., Bone L.R., Levine D. (2015). The Effect of a Community-Based Self-Help Intervention: Korean Americans With Type 2 Diabetes. Am. J. Prev. Med..

[83] Piette J.D., Richardson C., Himle J., Duffy S., Torres T., Vogel M., Barber K., Valenstein M. (2011). A randomized trial of telephonic counseling plus walking for depressed diabetes patients. Med. Care.

[84] Xu C., Dong Z., Zhang P., Chang G., Xiang Q., Zhang M., Zhou J., Qiao C., Yang Q., Qin Y. (2021). Effect of group cognitive behavioural therapy on psychological stress and blood glucose in people with type 2 diabetes mellitus: A community-based cluster randomized controlled trial in China. Diabet. Med..

[85] Zhang H.Z., Zhang P., Chang G.Q., Xiang Q.Y., Cao H., Zhou J.Y., Dong Z.M., Qiao C., Xu C.R., Qin Y. (2021). Effectiveness of cognitive behavior therapy for sleep disturbance and glycemic control in persons with type 2 diabetes mellitus: A community-based randomized controlled trial in China. World J. Diabetes.

[86] Kinmonth A.L., Woodcock A., Griffin S., Spiegal N., Campbell M.J. (1998). Randomised controlled trial of patient centred care of diabetes in general practice: Impact on current wellbeing and future disease risk. The Diabetes Care From Diagnosis Research Team. BMJ.

[87] Meulepas M.A., Braspenning J.C., de Grauw W.J., Lucas A.E., Wijkel D., Grol R.P. (2008). Patient-oriented intervention in addition to centrally organised checkups improves diabetic patient outcome in primary care. Qual. Saf. Health Care.

[88] Pill R., Stott N.C., Rollnick S.R., Rees M. (1998). A randomized controlled trial of an intervention designed to improve the care given in general practice to Type II diabetic patients: Patient outcomes and professional ability to change behaviour. Fam. Pr..

[89] Gallegos E.C., Ovalle-Berúmen F., Gomez-Meza M.V. (2006). Metabolic control of adults with type 2 diabetes mellitus through education and counseling. J. Nurs. Scholarsh..

[90] Ridgeway N.A., Harvill D.R., Harvill L.M., Falin T.M., Forester G.M., Gose O.D. (1999). Improved control of type 2 diabetes mellitus: A practical education/behavior modification program in a primary care clinic. South. Med. J..

[91] Whitehead L.C., Crowe M.T., Carter J.D., Maskill V.R., Carlyle D., Bugge C., Frampton C.M.A. (2017). A nurse-led education and cognitive behaviour therapy-based intervention among adults with uncontrolled type 2 diabetes: A randomised controlled trial. J. Eval. Clin. Pract..

[92] Chaves-Fonseca R.M., Matos O.S., Lordelo R.A., Abreu M., Farias M.G., Coutinho J.F., Ribeiro M.N., Matteoni-Athayde L., Lessa I., Pousada J. (2009). Implementation of a systematic approach to diabetes in primary care in Bahia, Brazil improves metabolic outcomes: PRODIBA-Programa de Interiorização da Assistência ao Diabetes na Bahia (Project for Dissemination of Diabetes Care in the State of Bahia). Diabet. Med..

[93] Frei A., Senn O., Chmiel C., Reissner J., Held U., Rosemann T. (2014). Implementation of the chronic care model in small medical practices improves cardiovascular risk but not glycemic control. Diabetes Care.

[94] Furler J., O’Neal D., Speight J., Manski-Nankervis J.A., Gorelik A., Holmes-Truscott E., Ginnivan L., Young D., Best J., Dean (2017). Supporting insulin initiation in type 2 diabetes in primary care: Results of the Stepping Up pragmatic cluster randomised controlled clinical trial. BMJ.

[95] Lou Q., Ye Q., Wu H., Wang Z., Ware R.S., Xiong Y., Xu F. (2020). Effectiveness of a clinic-based randomized controlled intervention for type 2 diabetes management: An innovative model of intensified diabetes management in Mainland China (C-IDM study). BMJ Open Diabetes Res. Care.

[96] Musacchio N., Lovagnini Scher A., Giancaterini A., Pessina L., Salis G., Schivalocchi F., Nicolucci A., Pellegrini F., Rossi M.C.E. (2011). Impact of a chronic care model based on patient empowerment on the management of Type2 diabetes: Effects of the SINERGIA programme. Diabet. Med..

[97] Panisch S., Johansson T., Flamm M., Winkler H., Weitgasser R., Sönnichsen A.C. (2018). The impact of a disease management programme for type 2 diabetes on health-related quality of life: Multilevel analysis of a cluster-randomised controlled trial. Diabetol. Metab. Syndr..

[98] Sönnichsen A.C., Winkler H., Flamm M., Panisch S., Kowatsch P., Klima G., Fürthauer B., Weitgasser R. (2010). The effectiveness of the Austrian disease management programme for type 2 diabetes: A cluster-randomised controlled trial. BMC Fam. Pr..

[99] Varroud-Vial M., Simon D., Attali J., Durand-Zaleski I., Bera L., Attali C., Letondeur C., Strauss K., Petit C., Charpentier G. (2004). Improving glycaemic control of patients with Type 2 diabetes in a primary care setting: A French application of the Staged Diabetes Management programme. Diabet. Med..

[100] Buhse S., Kuniss N., Liethmann K., Müller U.A., Lehmann T., Mühlhauser I. (2018). Informed shared decision-making programme for patients with type 2 diabetes in primary care: Cluster randomised controlled trial. BMJ Open.

[101] Mathers N., Ng C.J., Campbell M.J., Colwell B., Brown I., Bradley A. (2012). Clinical effectiveness of a patient decision aid to improve decision quality and glycaemic control in people with diabetes making treatment choices: A cluster randomised controlled trial (PANDAs) in general practice. BMJ Open.

[102] Wollny A., Altiner A., Daubmann A., Drewelow E., Helbig C., Löscher S., Pentzek M., Santos S., Wegscheider K., Wilm S. (2019). Patient-centered communication and shared decision making to reduce HbA1c levels of patients with poorly controlled type 2 diabetes mellitus—results of the cluster-randomized controlled DEBATE trial. BMC Fam. Pr..

[103] Wollny A., Löffler C., Drewelow E., Altiner A., Helbig C., Daubmann A., Wegscheider K., Löscher S., Pentzek M., Wilm S. (2021). Shared decision making and patient-centeredness for patients with poorly controlled type 2 diabetes mellitus in primary care—Results of the cluster-randomised controlled DEBATE trial. BMC Fam. Pract..

[104] Khan M.A., Walley J.D., Khan N., Hicks J., Ahmed M., Khan S.E., Khan M.A., Khan H.J., Harries A.D. (2018). Effectiveness of an integrated diabetes care package at primary healthcare facilities: A cluster randomised trial in Pakistan. BJGP Open.

[105] Krein S.L., Klamerus M.L., Vijan S., Lee J.L., Fitzgerald J.T., Pawlow A., Reeves P., Hayward R.A. (2004). Case management for patients with poorly controlled diabetes: A randomized trial. Am. J. Med..

[106] Luchsinger J.A., Palmas W., Teresi J.A., Silver S., Kong J., Eimicke J.P., Weinstock R.S., Shea S. (2011). Improved diabetes control in the elderly delays global cognitive decline. J. Nutr. Health Aging.

[107] Weitzman S., Greenfield S., Billimek J., Hava T., Schvartzman P., Yehiel E., Tandeter H., Eilat-Tsanani S., Kaplan S.H. (2009). Improving combined diabetes outcomes by adding a simple patient intervention to physician feedback: A cluster randomized trial. Isr. Med. Assoc. J..

[108] Allerton J., Mash R. (2020). The impact of intensified clinical care on glycaemic control in patients with type 2 diabetes at Khayelitsha Community Health Centre, South Africa: Quasi-experimental study. Prim. Care Diabetes.

[109] Bellary S., O’Hare J., Raymond N., Gumber A., Mughal S., Szczepura A., Kumar S., Barnett A. (2008). Enhanced diabetes care to patients of south Asian ethnic origin (the United Kingdom Asian Diabetes Study): A cluster randomised controlled trial. Lancet.

[110] Grant R.W., Wald J.S., Schnipper J.L., Gandhi T.K., Poon E.G., Orav E.J., Williams D.H., Volk L.A., Middleton B. (2008). Practice-linked online personal health records for type 2 diabetes mellitus: A randomized controlled trial. Arch. Intern. Med..

[111] Guldberg T.L., Vedsted P., Kristensen J.K., Lauritzen T. (2011). Improved quality of Type 2 diabetes care following electronic feedback of treatment status to general practitioners: A cluster randomized controlled trial. Diabet. Med..

[112] Hayashino Y., Suzuki H., Yamazaki K., Goto A., Izumi K., Noda M. (2016). A cluster randomized trial on the effect of a multifaceted intervention improved the technical quality of diabetes care by primary care physicians: The Japan Diabetes Outcome Intervention Trial-2 (J-DOIT2). Diabet. Med..

[113] Cleveringa F.G., Gorter K.J., van den Donk M., Pijman P.L., Rutten G.E. (2007). Task delegation and computerized decision support reduce coronary heart disease risk factors in type 2 diabetes patients in primary care. Diabetes Technol. Ther..

[114] Dale J., Caramlau I., Sturt J., Friede T., Walker R. (2009). Telephone peer-delivered intervention for diabetes motivation and support: The telecare exploratory RCT. Patient Educ. Couns..

[115] Denig P., Schuling J., Haaijer-Ruskamp F., Voorham J. (2014). Effects of a patient oriented decision aid for prioritising treatment goals in diabetes: Pragmatic randomised controlled trial. BMJ.

[116] Dijkstra R., Braspenning J., Grol R. (2008). Implementing diabetes passports to focus practice reorganization on improving diabetes care. Int. J. Qual. Health Care.

[117] Glasgow R.E., Nutting P.A., King D.K., Nelson C.C., Cutter G., Gaglio B., Rahm A.K., Whitesides H. (2005). Randomized effectiveness trial of a computer-assisted intervention to improve diabetes care. Diabetes Care.

[118] Holbrook A., Thabane L., Keshavjee K., Dolovich L., Bernstein B., Chan D., Troyan S., Foster G., Gerstein H. (2009). Individualized electronic decision support and reminders to improve diabetes care in the community: COMPETE II randomized trial. CMAJ.

[119] Jia W., Zhang P., Zhu D., Duolikun N., Li H., Bao Y., Li X. (2021). Evaluation of an mHealth-enabled hierarchical diabetes management intervention in primary care in China (ROADMAP): A cluster randomized trial. PLoS Med..

[120] Meulepas M.A., Braspenning J.C., de Grauw W.J., Lucas A.E., Harms L., Akkermans R.P., Grol R.P. (2007). Logistic support service improves processes and outcomes of diabetes care in general practice. Fam. Pr..

[121] Nesari M., Zakerimoghadam M., Rajab A., Bassampour S., Faghihzadeh S. (2010). Effect of telephone follow-up on adherence to a diabetes therapeutic regimen. Jpn. J. Nurs. Sci..

[122] O’Connor P.J., Sperl-Hillen J.M., Rush W.A., Johnson P.E., Amundson G.H., Asche S.E., Ekstrom H.L., Gilmer T.P. (2011). Impact of electronic health record clinical decision support on diabetes care: A randomized trial. Ann. Fam. Med..

[123] Powers B., Grubber J.M., Olsen M., Crowley M., Bosworth H. (2012). The cholesterol, hypertension, and glucose education (change) study: Results from a randomized controlled trial in African Americans with diabetes. J. Gen. Intern. Med..

[124] Ramallo-Fariña Y., García-Bello M.A., García-Pérez L., Boronat M., Wägner A.M., Rodríguez-Rodríguez L., de Pablos-Velasco P., Llorente Gómez de Segura I., González-Pacheco H., Carmona Rodríguez M. (2020). Effectiveness of Internet-Based Multicomponent Interventions for Patients and Health Care Professionals to Improve Clinical Outcomes in Type 2 Diabetes Evaluated Through the INDICA Study: Multiarm Cluster Randomized Controlled Trial. JMIR Mhealth Uhealth.

[125] Ramallo-Fariña Y., Rivero-Santana A., García-Pérez L., García-Bello M.A., Wägner A.M., Gonzalez-Pacheco H., Rodríguez-Rodríguez L., Kaiser-Girardot S., Monzón-Monzón G., Guerra-Marrero C. (2021). Patient-reported outcome measures for knowledge transfer and behaviour modification interventions in type 2 diabetes-the INDICA study: A multiarm cluster randomised controlled trial. BMJ Open.

[126] Sperl-Hillen J.M., O’Connor P.J., Rush W.A., Johnson P.E., Gilmer T., Biltz G., Asche S.E., Ekstrom H.L. (2010). Simulated physician learning program improves glucose control in adults with diabetes. Diabetes Care.

[127] Welch G., Allen N.A., Zagarins S.E., Stamp K.D., Bursell S.E., Kedziora R.J. (2011). Comprehensive diabetes management program for poorly controlled Hispanic type 2 patients at a community health center. Diabetes Educ..

[128] Young R.J., Taylor J., Friede T., Hollis S., Mason J.M., Lee P., Burns E., Long A.F., Gambling T., New J.P. (2005). Pro-active call center treatment support (PACCTS) to improve glucose control in type 2 diabetes: A randomized controlled trial. Diabetes Care.

[129] Carallo C., Scavelli F.B., Cipolla M., Merante V., Medaglia V., Irace C., Gnasso A. (2015). Management of type 2 diabetes mellitus through telemedicine. PLoS ONE.

[130] Cho J.-H., Kwon H.-S., Kim H.-S., Oh J.-A., Yoon K.-H. (2011). Effects on diabetes management of a health-care provider mediated, remote coaching system via a PDA-type glucometer and the Internet. J. Telemed. Telecare.

[131] Stone R.A., Rao R.H., Sevick M.A., Cheng C., Hough L.J., Macpherson D.S., Franko C.M., Anglin R.A., Obrosky D.S., Derubertis F.R. (2010). Active care management supported by home telemonitoring in veterans with type 2 diabetes: The DiaTel randomized controlled trial. Diabetes Care.

[132] Tang P.C., Overhage J.M., Chan A.S., Brown N.L., Aghighi B., Entwistle M.P., Hui S.L., Hyde S.M., Klieman L.H., Mitchell C.J. (2013). Online disease management of diabetes: Engaging and motivating patients online with enhanced resources-diabetes (EMPOWER-D), a randomized controlled trial. J. Am. Med. Inf. Assoc..

[133] Wakefield B.J., Holman J.E., Ray A., Scherubel M., Adams M.R., Hillis S.L., Rosenthal G.E. (2011). Effectiveness of home telehealth in comorbid diabetes and hypertension: A randomized, controlled trial. Telemed. J. E Health.

[134] Welch G., Zagarins S.E., Santiago-Kelly P., Rodriguez Z., Bursell S.E., Rosal M.C., Gabbay R.A. (2015). An internet-based diabetes management platform improves team care and outcomes in an urban Latino population. Diabetes Care.

[135] Wild S.H., Hanley J., Lewis S.C., McKnight J.A., McCloughan L.B., Padfield P.L., Parker R.A., Paterson M., Pinnock H., Sheikh A. (2016). Supported Telemonitoring and Glycemic Control in People with Type 2 Diabetes: The Telescot Diabetes Pragmatic Multicenter Randomized Controlled Trial. PLoS Med..

[136] Yang Y., Lee E.Y., Kim H.S., Lee S.H., Yoon K.H., Cho J.H. (2020). Effect of a Mobile Phone-Based Glucose-Monitoring and Feedback System for Type 2 Diabetes Management in Multiple Primary Care Clinic Settings: Cluster Randomized Controlled Trial. JMIR Mhealth Uhealth.

[137] Noda M., Hayashino Y., Yamazaki K., Suzuki H., Goto A., Kato M., Izumi K., Kobayashi M. (2020). A cluster-randomized trial of the effectiveness of a triple-faceted intervention promoting adherence to primary care physician visits by diabetes patients. Sci. Rep..

[138] Alonso-Domínguez R., Patino-Alonso M.C., Sánchez-Aguadero N., García-Ortiz L., Recio-Rodríguez J.I., Gómez-Marcos M.A. (2019). Effect of a multifactorial intervention on the increase in physical activity in subjects with type 2 diabetes mellitus: A randomized clinical trial (EMID Study). Eur. J. Cardiovasc. Nurs..

[139] Navicharern R., Aungsuroch Y., Thanasilp S. (2009). Effects of multifaceted nurse-coaching intervention on diabetic complications and satisfaction of persons with type 2 diabetes. J. Med. Assoc. Thail..

[140] van Bruggen R., Gorter K.J., Stolk R.P., Verhoeven R.P., Rutten G.E.H.M. (2008). Implementation of locally adapted guidelines on type 2 diabetes. Fam. Pract..

[141] Prestes M., Gayarre M.A., Elgart J.F., Gonzalez L., Rucci E., Paganini J.M., Gagliardino J.J. (2017). Improving diabetes care at primary care level with a multistrategic approach: Results of the DIAPREM programme. Acta Diabetol..

[142] Peterson K.A., Radosevich D.M., O’Connor P.J., Nyman J.A., Prineas R.J., Smith S.A., Arneson T.J., Corbett V.A., Weinhandl J.C., Lange C.J. (2008). Improving Diabetes Care in Practice: Findings from the TRANSLATE trial. Diabetes Care.

[143] Olivarius N.D.F., Beck-Nielsen H., Andreasen A.H., Hørder M., Pedersen P.A. (2001). Randomised controlled trial of structured personal care of type 2 diabetes mellitus. Br. Med. J..

[144] Ong K.L., Stafford L.K., McLaughlin S.A., Boyko E.J., Vollset S.E., Smith A.E., Dalton B.E., Duprey J., Cruz J.A., Hagins H. (2023). Global, regional, and national burden of diabetes from 1990 to 2021, with projections of prevalence to 2050: A systematic analysis for the Global Burden of Disease Study 2021. Lancet.

[145] Murthy S., Kamath P., Godinho M.A., Gudi N., Jacob A., John O. (2023). Digital health innovations for non-communicable disease management during the COVID-19 pandemic: A rapid scoping review. BMJ Innov..

[146] Flood D., Seiglie J.A., Dunn M., Tschida S., Theilmann M., Marcus M.E., Brian G., Norov B., Mayige M.T., Singh Gurung M. (2021). The state of diabetes treatment coverage in 55 low-income and middle-income countries: A cross-sectional study of nationally representative, individual-level data in 680 102 adults. Lancet Healthy Longev..

[147] Agarwal S., Wade A.N., Mbanya J.C., Yajnik C., Thomas N., Egede L.E., Campbell J.A., Walker R.J., Maple-Brown L., Graham S. (2023). The role of structural racism and geographical inequity in diabetes outcomes. Lancet.

[148] Greaves C.J., Middlebrooke A., O’Loughlin L., Holland S., Piper J., Steele A., Gale T., Hammerton F., Daly M. (2008). Motivational interviewing for modifying diabetes risk: A randomised controlled trial. Br. J. Gen. Pr..

[149] Bilgin A., Muz G., Yuce G.E. (2022). The effect of motivational interviewing on metabolic control and psychosocial variables in individuals diagnosed with diabetes: Systematic review and meta-analysis. Patient Educ. Couns..

[150] Duke S.-A.S., Colagiuri S., Colagiuri R. (2009). Individual patient education for people with type 2 diabetes mellitus. Cochrane Database Syst. Rev..

[151] Christie D., Channon S. (2014). The potential for motivational interviewing to improve outcomes in the management of diabetes and obesity in paediatric and adult populations: A clinical review. Diabetes Obes. Metab..

[152] Sigurdardottir A.K., Jonsdottir H., Benediktsson R. (2007). Outcomes of educational interventions in type 2 diabetes: WEKA data-mining analysis. Patient Educ. Couns..

[153] Ekong G., Kavookjian J. (2016). Motivational interviewing and outcomes in adults with type 2 diabetes: A systematic review. Patient Educ. Couns..

[154] Umpierre D., Ribeiro P.A.B., Kramer C.K., Leitão C.B., Zucatti A.T.N., Azevedo M.J., Gross J.L., Ribeiro J.P., Schaan B.D. (2011). Physical activity advice only or structured exercise training and association with HbA1c levels in type 2 diabetes: A systematic review and meta-analysis. JAMA.

[155] Palma-Duran S.A., Vlassopoulos A., Lean M., Govan L., Combet E. (2017). Nutritional intervention and impact of polyphenol on glycohemoglobin (HbA1c) in non-diabetic and type 2 diabetic subjects: Systematic review and meta-analysis. Crit. Rev. Food Sci. Nutr..

[156] Xiong S., Lu H., Peoples N., Duman E.K., Najarro A., Ni Z., Gong E., Yin R., Ostbye T., Palileo-Villanueva L.M. (2023). Digital health interventions for non-communicable disease management in primary health care in low-and middle-income countries. npj Digit. Med..

[157] Fu Y., Nelson E.A., McGowan L. (2019). Multifaceted self-management interventions for older women with urinary incontinence: A systematic review and narrative synthesis. BMJ Open.

[158] Williams J.W., Gerrity M., Holsinger T., Dobscha S., Gaynes B., Dietrich A. (2007). Systematic review of multifaceted interventions to improve depression care. Gen. Hosp. Psychiatry.

[159] Cleveringa F.G.W., Welsing P.M.J., van den Donk M., Gorter K.J., Niessen L.W., Rutten G.E.H.M., Redekop W.K. (2010). Cost-effectiveness of the diabetes care protocol, a multifaceted computerized decision support diabetes management intervention that reduces cardiovascular risk. Diabetes Care.

[160] Effective Practice Organisation of Care EPOC Taxonomy. https://epoc.cochrane.org/epoc-taxonomy.

[161] Pimouguet C., Goff M.L., Thiébaut R., Dartigues J.F., Helmer C. (2011). Effectiveness of disease-management programs for improving diabetes care: A meta-analysis. CMAJ.

[162] Knight K., Badamgarav E., Henning J.M., Hasselblad V., Gano A.D., Ofman J.J., Weingarten S.R. (2005). A systematic review of diabetes disease management programs. Am. J. Manag. Care.

[163] Davies M.J., Aroda V.R., Collins B.S., Gabbay R.A., Green J., Maruthur N.M., Rosas S.E., Del Prato S., Mathieu C., Mingrone G. (2022). Management of Hyperglycemia in Type 2 Diabetes, 2022. A Consensus Report by the American Diabetes Association (ADA) and the European Association for the Study of Diabetes (EASD). Diabetes Care.

[164] Rodriguez-Gutierrez R., Gionfriddo M.R., Ospina N.S., Maraka S., Tamhane S., Montori V.M., Brito J.P. (2016). Shared decision making in endocrinology: Present and future directions. Lancet Diabetes Endocrinol..

[165] Kruse C.S., Stein A., Thomas H., Kaur H. (2018). The use of Electronic Health Records to Support Population Health: A Systematic Review of the Literature. J. Med. Syst..

[166] Shan R., Sarkar S., Martin S.S. (2019). Digital health technology and mobile devices for the management of diabetes mellitus: State of the art. Diabetologia.

[167] Zhai Y.-k., Zhu W.-j., Cai Y.-l., Sun D.-x., Zhao J. (2014). Clinical- and Cost-effectiveness of Telemedicine in Type 2 Diabetes Mellitus: A Systematic Review and Meta-analysis. Medicine.

[168] Lee J.Y., Lee S.W.H. (2018). Telemedicine Cost–Effectiveness for Diabetes Management: A Systematic Review. Diabetes Technol. Ther..

[169] Dixit S., Nandakumar G. (2021). Promoting healthy lifestyles using information technology during the COVID-19 pandemic. RCM.

[170] Hollander J.E., Carr B.G. (2020). Virtually Perfect? Telemedicine for COVID-19. N. Engl. J. Med..

[171] Rao K.D., Mehta A., Kautsar H., Kak M., Karem G., Misra M., Joshi H., Herbst C.H., Perry H.B. (2023). Improving quality of non-communicable disease services at primary care facilities in middle-income countries: A scoping review. Soc. Sci. Med..

[172] Stotz S.A., McNealy K., Begay R.L., DeSanto K., Manson S.M., Moore K.R. (2021). Multi-level Diabetes Prevention and Treatment Interventions for Native People in the USA and Canada: A Scoping Review. Curr. Diabetes Rep..

[173] Chen M., Wang L., Chen W., Zhang L., Jiang H., Mao W. (2014). Does economic incentive matter for rational use of medicine? China’s experience from the essential medicines program. Pharmacoeconomics.

[174] Rowe A.K., Rowe S.Y., Peters D.H., Holloway K.A., Chalker J., Ross-Degnan D. (2018). Effectiveness of strategies to improve health-care provider practices in low-income and middle-income countries: A systematic review. Lancet Glob. Health.

[175] Nishijima M., Sarti F.M., Vodenska I., Zhang G. (2019). Effects of decentralization of primary health care on diabetes mellitus in Brazil. Public Health.

[176] Ricci-Cabello I., Carvallo-Castañeda D., Vásquez-Mejía A., Alonso-Coello P., Saz-Parkinson Z., Parmelli E., Morgano G.P., Rigau D., Solà I., Neamtiu L. (2023). Characteristics and impact of interventions to support healthcare providers’ compliance with guideline recommendations for breast cancer: A systematic literature review. Implement. Sci..

