# Supplementary material for: Enhancing Type 2 Diabetes Management Involving Healthcare Professionals in Primary Care
Source: Healthcare (Basel). 2026 Aug 20;14(16):2633. doi: 10.3390/healthcare14162633 (PMC13512607; doi:10.3390/healthcare14162633)
Supplement: Supplementary file 1 [file healthcare-14-02633-s001.zip › healthcare-4408098-supplementary.pdf]

Review

Enhancing type 2 diabetes management involving healthcare professionals in primary care

Mengyao Li and Lijian Wang *

Supplemental Information

Table S1: Search strategy in CENTRAL

Table S2: Search strategy in MEDLINE

Table S3: Search strategy in Embase

Table S4: Search strategy in PsycINFO

Table S5: Search strategy in CINAHL

Table S6: Basic characteristics of the interventions in the 112 included studies

Table S1: Search strategy in CENTRAL

Search number	Query	Results
#1	MeSH descriptor: [Diabetes Mellitus, Type 2] explode all trees	19,976
#2	(“diabetes mellitus, noninsulin dependent” OR “ketosis resistant diabetes mellitus” OR “diabetes mellitus, non insulin dependent” OR “non insulin dependent diabetes mellitus” OR “diabetes mellitus, stable” OR “stable diabetes mellitus” OR “NIDDM” OR “diabetes mellitus, noninsulin dependent” OR “diabetes mellitus, maturity onset” OR “maturity onset diabetes mellitus” OR “MODY” OR “diabetes mellitus, slow onset” OR “type 2 diabetes mellitus” OR “noninsulin dependent diabetes mellitus” OR “diabetes, maturity onset” OR “maturity onset diabetes” OR “type 2 diabetes” OR “diabetes, type 2” OR “adult onset diabetes mellitus” OR “diabetes mellitus, adult onset”):ti,ab,kw	40,940
#3	MeSH descriptor: [Primary Health Care] explode all trees	8,364
#4	MeSH descriptor: [Ambulatory Care] explode all trees	3,753
#5	MeSH descriptor: [Community Health Services] explode all trees	15,089
#6	MeSH descriptor: [Group Practice] explode all trees	332
#7	MeSH descriptor: [Community Health Centers] explode all trees	590
#8	MeSH descriptor: [General Practice] explode all trees	2,504
#9	(“Care, Primary Health” OR “Health Care, Primary” OR “Primary Healthcare” OR “Healthcare, Primary” OR “Primary Care” OR “Care, Primary” OR “Care, Ambulatory” OR “Outpatient Care” OR “Care, Outpatient” OR “Health Services, Outpatient” OR “Health Service, Outpatient” OR “Outpatient Health Service” OR “Outpatient Health Services” OR “Outpatient Services” OR “Outpatient Service” OR “Service, Outpatient” OR “Services, Outpatient” OR “Services, Outpatient Health” OR “Clinic Visits” OR “Clinic Visit” OR “Visit, Clinic” OR “Visits, Clinic” OR “Community Health Service” OR “Health Service, Community” OR “Service, Community Health” OR “Services, Community Health” OR “Health Services, Community” OR “Community Health Care” OR “Care, Community Health” OR “Health Care, Community” OR “Community Healthcare” OR “Healthcare, Community” OR “Practice, General” OR “General practices” OR “Group Practices” OR “Practice, Group” OR “Practices, Group” OR “Center, Community Health” OR “Centers, Community Health” OR “Community Health Center” OR “Health Center, Community” OR “Health Centers, Community” OR “Neighborhood Health Centers” OR “Health Centers, Neighborhood” OR “Neighborhood Health Center” OR clinic? OR practice? OR community OR communities OR family OR families OR township? OR village? OR “health station” OR “health stations”):ti,ab,kw	230,833
#10	MeSH descriptor: [Crisis Intervention] explode all trees	224
#11	MeSH descriptor: [Psychosocial Intervention] explode all trees	105
#12	MeSH descriptor: [Internet-Based Intervention] explode all trees	351
#13	MeSH descriptor: [Early Medical Intervention] explode all trees	426
#14	(intervention* OR implementation* OR program*):ti,ab,kw	536,542
#15	(“randomized controlled trial” OR “randomized controlled trials” OR random* OR “quasi experiment study” OR “quasi experiment studies” OR “quasi NEXT experiment*” OR “quasiexperiment*” OR (pretest near/4 posttest) OR (pre near/4 post) OR “nonequivalent NEXT group*” OR “natural NEXT experiment*” OR “regression discontinuity” OR (before near/4 after) OR “interrupted time series”):ti,ab,kw	1,182,145
#16	#1 OR #2	44,893

#17	#3 OR #4 OR #5 OR #6 OR #7 OR #8 OR #9	241,130
#18	#10 OR #11 OR #12 OR #13 OR #14	536,542
#19	#15 AND #16 AND #17 AND #18	4,037

Table S2: Search strategy in MEDLINE

Search number	Query	Results
1	"diabetes mellitus, type 2"[MeSH Terms] OR "diabetes mellitus, noninsulin dependent"[Title/Abstract] OR "ketosis resistant diabetes mellitus"[Title/Abstract] OR "diabetes mellitus, non insulin dependent"[Title/Abstract] OR "non insulin dependent diabetes mellitus"[Title/Abstract] OR "diabetes mellitus, stable"[Title/Abstract] OR "stable diabetes mellitus"[Title/Abstract] OR "NIDDM"[Title/Abstract] OR "diabetes mellitus, noninsulin dependent"[Title/Abstract] OR "diabetes mellitus, maturity onset"[Title/Abstract] OR "maturity onset diabetes mellitus"[Title/Abstract] OR "MODY"[Title/Abstract] OR "diabetes mellitus, slow onset"[Title/Abstract] OR "type 2 diabetes mellitus"[Title/Abstract] OR "noninsulin dependent diabetes mellitus"[Title/Abstract] OR "diabetes, maturity onset"[Title/Abstract] OR "maturity onset diabetes"[Title/Abstract] OR "type 2 diabetes"[Title/Abstract] OR "diabetes, type 2"[Title/Abstract] OR "adult onset diabetes mellitus"[Title/Abstract] OR "diabetes mellitus, adult onset"[Title/Abstract]	215,521
2	"primary health care"[MeSH Terms] OR "ambulatory care"[MeSH Terms] OR "community health services"[MeSH Terms] OR "general practice"[MeSH Terms] OR "group practice"[MeSH Terms] OR "community health centers"[MeSH Terms] OR "Care, Primary Health"[Title/Abstract] OR "Health Care, Primary"[Title/Abstract] OR "Primary Healthcare"[Title/Abstract] OR "Healthcare, Primary"[Title/Abstract] OR "Primary Care"[Title/Abstract] OR "Care, Primary"[Title/Abstract] OR "Care, Ambulatory"[Title/Abstract] OR "Outpatient Care"[Title/Abstract] OR "Care, Outpatient"[Title/Abstract] OR "Health Services, Outpatient"[Title/Abstract] OR "Health Service, Outpatient"[Title/Abstract] OR "Outpatient Health Service"[Title/Abstract] OR "Outpatient Health Services"[Title/Abstract] OR "Outpatient Services"[Title/Abstract] OR "Outpatient Service"[Title/Abstract] OR "Service, Outpatient"[Title/Abstract] OR "Services, Outpatient"[Title/Abstract] OR "Services, Outpatient Health"[Title/Abstract] OR "Clinic Visits"[Title/Abstract] OR "Clinic Visit"[Title/Abstract] OR "Visit, Clinic"[Title/Abstract] OR "Visits, Clinic"[Title/Abstract] OR "Community Health Service"[Title/Abstract] OR "Health Service, Community"[Title/Abstract] OR "Service, Community Health"[Title/Abstract] OR "Services, Community Health"[Title/Abstract] OR "Health Services, Community"[Title/Abstract] OR "Community Health Care"[Title/Abstract] OR "Care, Community Health"[Title/Abstract] OR "Health Care, Community"[Title/Abstract] OR "Community Healthcare"[Title/Abstract] OR "Healthcare, Community"[Title/Abstract] OR "Practice, General"[Title/Abstract] OR "General practices"[Title/Abstract] OR "Group Practices"[Title/Abstract] OR "Practice, Group"[Title/Abstract] OR "Practices, Group"[Title/Abstract] OR "Center, Community Health"[Title/Abstract] OR "Centers, Community Health"[Title/Abstract] OR "Community Health Center"[Title/Abstract] OR "Health Center, Community"[Title/Abstract] OR "Health Centers, Community"[Title/Abstract] OR "Neighborhood Health Centers"[Title/Abstract] OR "Health Centers, Neighborhood"[Title/Abstract] OR "Neighborhood Health Center"[Title/Abstract] OR clinic[Title/Abstract] OR clinics[Title/Abstract] OR practice[Title/Abstract] OR	3,273,441

	practices[Title/Abstract] OR community[Title/Abstract] OR communities[Title/Abstract] OR family[Title/Abstract] OR families[Title/Abstract] OR township[Title/Abstract] OR townships[Title/Abstract] OR village[Title/Abstract] OR villages[Title/Abstract] OR "health station" [Title/Abstract] OR "health stations" [Title/Abstract]	
3	"crisis Intervention"[MeSH Terms] OR "psychosocial intervention"[MeSH Terms] OR "internet-based intervention"[MeSH Terms] OR "early medical intervention"[MeSH Terms] OR intervention*[Title/Abstract] OR implementation*[Title/Abstract] OR program*[Title/Abstract]	2,270,384
4	"randomized controlled trial"[Title/Abstract] OR "randomized controlled trial"[Title/Abstract] OR "random*"[Title/Abstract] OR "quasi experiment study"[Title/Abstract] OR "quasi experiment*"[Title/Abstract] OR "quasiexperiment*"[Title/Abstract] OR "pretest posttest"[Title/Abstract] OR "pre post"[Title/Abstract] OR "nonequivalent group"[Title/Abstract] OR "nonequivalent groups"[Title/Abstract] OR "natural experiment*"[Title/Abstract] OR "regression discontinuity"[Title/Abstract] OR "before after"[Title/Abstract] OR "interrupted time series"[Title/Abstract]	1,366,672
5	#1 AND #2 AND #3 AND #4	3,199

Table S3: Search strategy in Embase

Search number	Query	Results
#1	'diabetes mellitus, type 2'/exp OR 'diabetes mellitus, type 2' OR 'ketosis resistant diabetes mellitus':ab,ti OR 'diabetes mellitus, non insulin dependent':ab,ti OR 'non insulin dependent diabetes mellitus':ab,ti OR 'diabetes mellitus, stable':ab,ti OR 'stable diabetes mellitus':ab,ti OR 'niddm':ab,ti OR 'diabetes mellitus, noninsulin dependent':ab,ti OR 'diabetes mellitus, maturity onset':ab,ti OR 'maturity onset diabetes mellitus':ab,ti OR 'mody':ab,ti OR 'diabetes mellitus, slow onset':ab,ti OR 'type 2 diabetes mellitus':ab,ti OR 'noninsulin dependent diabetes mellitus':ab,ti OR 'diabetes, maturity onset':ab,ti OR 'maturity onset diabetes':ab,ti OR 'type 2 diabetes':ab,ti OR 'diabetes, type 2':ab,ti OR 'adult onset diabetes mellitus':ab,ti OR 'diabetes mellitus, adult onset':ab,ti	348474
#2	'primary health care'/exp OR 'ambulatory care'/exp OR 'community health services'/exp OR 'general practice'/exp OR 'group practice'/exp OR 'community health centers'/exp OR 'care, primary health':ab,ti OR 'health care, primary':ab,ti OR 'primary healthcare':ab,ti OR 'healthcare, primary':ab,ti OR 'primary care':ab,ti OR 'care, primary':ab,ti OR 'care, ambulatory':ab,ti OR 'outpatient care':ab,ti OR 'care, outpatient':ab,ti OR 'health services, outpatient':ab,ti OR 'health service, outpatient':ab,ti OR 'outpatient health service':ab,ti OR 'outpatient health services':ab,ti OR 'outpatient services':ab,ti OR 'outpatient service':ab,ti OR 'service, outpatient':ab,ti OR 'services, outpatient':ab,ti OR 'services, outpatient health':ab,ti OR 'clinic visits':ab,ti OR 'clinic visit':ab,ti OR 'visit, clinic':ab,ti OR 'visits, clinic':ab,ti OR 'community health service':ab,ti OR 'health service, community':ab,ti OR 'service, community health':ab,ti OR 'services, community health':ab,ti OR 'health services, community':ab,ti OR 'community health care':ab,ti OR 'care, community health':ab,ti OR 'health care, community':ab,ti OR 'community healthcare':ab,ti OR 'healthcare, community':ab,ti OR 'practice, general':ab,ti OR 'general practices':ab,ti OR 'group practices':ab,ti OR 'practice, group':ab,ti OR 'practices, group':ab,ti OR 'center, community health':ab,ti OR 'centers, community health':ab,ti OR 'community health center':ab,ti OR 'health center, community':ab,ti OR 'health centers, community':ab,ti OR 'neighborhood health centers':ab,ti OR 'health centers, neighborhood':ab,ti OR 'neighborhood health center':ab,ti OR clinic$:ab,ti OR practice$:ab,ti OR communit*:ab,ti OR township$:ab,ti OR village$:ab,ti OR ‘health station$’:ab,ti OR family:ab,ti OR families:ab,ti	4084958
#3	'crisis intervention'/exp OR 'psychosocial intervention'/exp OR 'internet-based intervention'/exp OR 'early medical intervention'/exp OR 'intervention*':ab,ti OR 'implementation*':ab,ti OR 'program*':ab,ti	3037902
#4	'randomized controlled trial$':ab,ti OR 'random*':ab,ti OR 'quasi experiment study':ab,ti OR 'quasi experiment studies':ab,ti OR 'quasiexperiment*':ab,ti OR 'quasi experiment*':ab,ti OR (('pretest' NEAR/4 'posttest'):ab,ti) OR (('pre' NEAR/4 'post'):ab,ti) OR 'nonequivalent group$':ab,ti OR 'natural experiment*':ab,ti OR 'regression discontinuity':ab,ti OR (('before' NEAR/4 'after'):ab,ti) OR 'interrupted time series':ab,ti	2448385
#5	#1 AND #2 AND #3 AND #4	4482

Table S4: Search strategy in PsycINFO

Search number	Query	Results
S1	TI (“diabetes mellitus, type 2” OR “diabetes mellitus, noninsulin dependent” OR “ketosis resistant diabetes mellitus” OR “diabetes mellitus, non insulin dependent” OR “non insulin dependent diabetes mellitus” OR “diabetes mellitus, stable” OR “stable diabetes mellitus” OR “NIDDM” OR “diabetes mellitus, noninsulin dependent” OR “diabetes mellitus, maturity onset” OR “maturity onset diabetes mellitus” OR “MODY” OR “diabetes mellitus, slow onset” OR “type 2 diabetes mellitus” OR “noninsulin dependent diabetes mellitus” OR “diabetes, maturity onset” OR “maturity onset diabetes” OR “type 2 diabetes” OR “diabetes, type 2” OR “adult onset diabetes mellitus” OR “diabetes mellitus, adult onset”) OR AB (“diabetes mellitus, type 2” OR “diabetes mellitus, noninsulin dependent” OR “ketosis resistant diabetes mellitus” OR “diabetes mellitus, non insulin dependent” OR “non insulin dependent diabetes mellitus” OR “diabetes mellitus, stable” OR “stable diabetes mellitus” OR “NIDDM” OR “diabetes mellitus, noninsulin dependent” OR “diabetes mellitus, maturity onset” OR “maturity onset diabetes mellitus” OR “MODY” OR “diabetes mellitus, slow onset” OR “type 2 diabetes mellitus” OR “noninsulin dependent diabetes mellitus” OR “diabetes, maturity onset” OR “maturity onset diabetes” OR “type 2 diabetes” OR “diabetes, type 2” OR “adult onset diabetes mellitus” OR “diabetes mellitus, adult onset”)	8,116
S2	TI (“primary health care” OR “ambulatory care” OR “community health services” OR “general practice” OR “group practice” OR “community health centers” OR “Care, Primary Health” OR “Health Care, Primary” OR “Primary Healthcare” OR “Healthcare, Primary” OR “Primary Care” OR “Care, Primary” OR “Care, Ambulatory” OR “Outpatient Care” OR “Care, Outpatient” OR “Health Services, Outpatient” OR “Health Service, Outpatient” OR “Outpatient Health Service” OR “Outpatient Health Services” OR “Outpatient Services” OR “Outpatient Service” OR “Service, Outpatient” OR “Services, Outpatient” OR “Services, Outpatient Health” OR “Clinic Visits” OR “Clinic Visit” OR “Visit, Clinic” OR “Visits, Clinic” OR “Community Health Service” OR “Health Service, Community” OR “Service, Community Health” OR “Services, Community Health” OR “Health Services, Community” OR “Community Health Care” OR “Care, Community Health” OR “Health Care, Community” OR “Community Healthcare” OR “Healthcare, Community” OR “Practice, General” OR “General practices” OR “Group Practices” OR “Practice, Group” OR “Practices, Group” OR “Center, Community Health” OR “Centers, Community Health” OR “Community Health Center” OR “Health Center, Community” OR “Health Centers, Community” OR “Neighborhood Health Centers” OR “Health Centers, Neighborhood” OR “Neighborhood Health Center” OR clinic# OR practice3 OR communit* OR township# OR village# OR “health station#” OR family OR families) OR AB (“primary health care” OR “ambulatory care” OR “community health services” OR “general practice” OR “group practice” OR “community health centers” OR “Care, Primary Health” OR “Health Care, Primary” OR “Primary Healthcare” OR “Healthcare, Primary” OR “Primary Care” OR “Care, Primary” OR “Care, Ambulatory” OR “Outpatient Care” OR “Care, Outpatient” OR “Health Services, Outpatient” OR “Health Service, Outpatient” OR “Outpatient Health Service” OR “Outpatient Health Services” OR “Outpatient Services” OR “Outpatient Service” OR “Service, Outpatient” OR “Services, Outpatient” OR “Services, Outpatient Health” OR “Clinic Visits” OR “Clinic Visit” OR “Visit, Clinic” OR “Visits, Clinic” OR “Community Health Service” OR “Health Service, Community”	761,040

	OR “Service, Community Health” OR “Services, Community Health” OR “Health Services, Community” OR “Community Health Care” OR “Care, Community Health” OR “Health Care, Community” OR “Community Healthcare” OR “Healthcare, Community” OR “Practice, General” OR “General practices” OR “Group Practices” OR “Practice, Group” OR “Practices, Group” OR “Center, Community Health” OR “Centers, Community Health” OR “Community Health Center” OR “Health Center, Community” OR “Health Centers, Community” OR “Neighborhood Health Centers” OR “Health Centers, Neighborhood” OR “Neighborhood Health Center” OR clinic# OR practice3 OR communit* OR township# OR village# OR “health station#” OR family OR families)	
S3	TI (“crisis Intervention” OR “psychosocial intervention” OR “internet-based intervention” OR “early medical intervention” OR “intervention*” OR “implementation*” OR “program*”) OR AB (“crisis Intervention” OR “psychosocial intervention” OR “internet-based intervention” OR “early medical intervention” OR “intervention*” OR “implementation*” OR “program*”)	823,740
S4	TI (“randomized controlled trial#” OR “random*” OR “quasi experiment study” OR “quasi experiment studies” OR “quasiexperiment*” OR “quasi experiment*” OR (pretest W3 posttest) OR (pre W3 post) OR “nonequivalent group#” OR “natural experiment#” OR “regression discontinuity” OR (before W3 after) OR “interrupted time series”) OR AB (“randomized controlled trial#” OR “random*” OR “quasi experiment study” OR “quasi experiment studies” OR “quasiexperiment*” OR “quasi experiment*” OR (pretest W3 posttest) OR (pre W3 post) OR “nonequivalent group#” OR “natural experiment#” OR “regression discontinuity” OR (before W3 after) OR “interrupted time series”)	321,162
S5	S1 AND S2 AND S3 AND S4	424

Table S5: Search strategy in CINAHL

Search number	Query	Results
S1	TI (“diabetes mellitus, type 2” OR “diabetes mellitus, noninsulin dependent” OR “ketosis resistant diabetes mellitus” OR “diabetes mellitus, non insulin dependent” OR “non insulin dependent diabetes mellitus” OR “diabetes mellitus, stable” OR “stable diabetes mellitus” OR “NIDDM” OR “diabetes mellitus, noninsulin dependent” OR “diabetes mellitus, maturity onset” OR “maturity onset diabetes mellitus” OR “MODY” OR “diabetes mellitus, slow onset” OR “type 2 diabetes mellitus” OR “noninsulin dependent diabetes mellitus” OR “diabetes, maturity onset” OR “maturity onset diabetes” OR “type 2 diabetes” OR “diabetes, type 2” OR “adult onset diabetes mellitus” OR “diabetes mellitus, adult onset”) OR AB (“diabetes mellitus, type 2” OR “diabetes mellitus, noninsulin dependent” OR “ketosis resistant diabetes mellitus” OR “diabetes mellitus, non insulin dependent” OR “non insulin dependent diabetes mellitus” OR “diabetes mellitus, stable” OR “stable diabetes mellitus” OR “NIDDM” OR “diabetes mellitus, noninsulin dependent” OR “diabetes mellitus, maturity onset” OR “maturity onset diabetes mellitus” OR “MODY” OR “diabetes mellitus, slow onset” OR “type 2 diabetes mellitus” OR “noninsulin dependent diabetes mellitus” OR “diabetes, maturity onset” OR “maturity onset diabetes” OR “type 2 diabetes” OR “diabetes, type 2” OR “adult onset diabetes mellitus” OR “diabetes mellitus, adult onset”)	59,303
S2	TI (“primary health care” OR “ambulatory care” OR “community health services” OR “general practice” OR “group practice” OR “community health centers” OR “Care, Primary Health” OR “Health Care, Primary” OR “Primary Healthcare” OR “Healthcare, Primary” OR “Primary Care” OR “Care, Primary” OR “Care, Ambulatory” OR “Outpatient Care” OR “Care, Outpatient” OR “Health Services, Outpatient” OR “Health Service, Outpatient” OR “Outpatient Health Service” OR “Outpatient Health Services” OR “Outpatient Services” OR “Outpatient Service” OR “Service, Outpatient” OR “Services, Outpatient” OR “Services, Outpatient Health” OR “Clinic Visits” OR “Clinic Visit” OR “Visit, Clinic” OR “Visits, Clinic” OR “Community Health Service” OR “Health Service, Community” OR “Service, Community Health” OR “Services, Community Health” OR “Health Services, Community” OR “Community Health Care” OR “Care, Community Health” OR “Health Care, Community” OR “Community Healthcare” OR “Healthcare, Community” OR “Practice, General” OR “General practices” OR “Group Practices” OR “Practice, Group” OR “Practices, Group” OR “Center, Community Health” OR “Centers, Community Health” OR “Community Health Center” OR “Health Center, Community” OR “Health Centers, Community” OR “Neighborhood Health Centers” OR “Health Centers, Neighborhood” OR “Neighborhood Health Center” OR clinic# OR practice3 OR communit* OR township# OR village# OR “health station#” OR family OR families) OR AB (“primary health care” OR “ambulatory care” OR “community health services” OR “general practice” OR “group practice” OR “community health centers” OR “Care, Primary Health” OR “Health Care, Primary” OR “Primary Healthcare” OR “Healthcare, Primary” OR “Primary Care” OR “Care, Primary” OR “Care, Ambulatory” OR “Outpatient Care” OR “Care, Outpatient” OR “Health Services, Outpatient” OR “Health Service, Outpatient” OR “Outpatient Health Service” OR “Outpatient Health Services” OR “Outpatient Services” OR “Outpatient Service” OR “Service, Outpatient” OR “Services, Outpatient” OR “Services, Outpatient Health” OR “Clinic Visits” OR “Clinic Visit” OR “Visit, Clinic” OR “Visits, Clinic” OR “Community Health Service” OR “Health Service, Community”	728,198

	OR “Service, Community Health” OR “Services, Community Health” OR “Health Services, Community” OR “Community Health Care” OR “Care, Community Health” OR “Health Care, Community” OR “Community Healthcare” OR “Healthcare, Community” OR “Practice, General” OR “General practices” OR “Group Practices” OR “Practice, Group” OR “Practices, Group” OR “Center, Community Health” OR “Centers, Community Health” OR “Community Health Center” OR “Health Center, Community” OR “Health Centers, Community” OR “Neighborhood Health Centers” OR “Health Centers, Neighborhood” OR “Neighborhood Health Center” OR clinic# OR practice3 OR communit* OR township# OR village# OR “health station#” OR family OR families)	
S3	TI (“crisis Intervention” OR “psychosocial intervention” OR “internet-based intervention” OR “early medical intervention” OR “intervention*” OR “implementation*” OR “program*”) OR AB (“crisis Intervention” OR “psychosocial intervention” OR “internet-based intervention” OR “early medical intervention” OR “intervention*” OR “implementation*” OR “program*”)	915,279
S4	TI (“randomized controlled trial#” OR “random*” OR “quasi experiment study” OR “quasi experiment studies” OR “quasiexperiment*” OR “quasi experiment*” OR (pretest W3 posttest) OR (pre W3 post) OR “nonequivalent group#” OR “natural experiment#” OR “regression discontinuity” OR (before W3 after) OR “interrupted time series”) OR AB (“randomized controlled trial#” OR “random*” OR “quasi experiment study” OR “quasi experiment studies” OR “quasiexperiment*” OR “quasi experiment*” OR (pretest W3 posttest) OR (pre W3 post) OR “nonequivalent group#” OR “natural experiment#” OR “regression discontinuity” OR (before W3 after) OR “interrupted time series”)	525,390
S5	S1 AND S2 AND S3 AND S4	1,264

Table S6: Basic characteristics of the interventions in the 112 included studies

No.	Study	Study design	Country/region	Continent	Income level	Provider	Intervention content	Intervention subcategory	Intervention category
1	(Akturan, Kaya et al. 2017)	Cluster randomized controlled trial	Turkey	Asia	Upper-middle-income economies	Physicians	Interview technique: Provider training	Interview	2 What care is provided
2	(Al Asmary, Al-Harbi et al. 2013)	Before-and-after study	Saudi Arabia	Asia	High-income economies	Team: a senior family physician; nurse; clinical pharmacist specialist; Dietitian; diabetic educator; health educator and social worker.	Integrated care program: Multidisciplinary team care	Multidisciplinary	1 Who provides care
3	(Allerton and Mash 2020)	Quasi-experimental study	South Africa	Africa	Upper-middle-income economies	Clinicians	Intensive care: Medication review or intensification; more frequent follow-up	Intensive care	3 How care is delivered
4	(Alonso-Domínguez, Patino-Alonso et al. 2019)	Randomized controlled trial	Spain	Europe	High-income economies	Nurses	Multifactorial intervention: Diet and physical-activity support; web/mobile support	Multifaceted	5 Multifaceted
5	(Bellary, O'Hare et al. 2008)	Cluster randomized controlled trial	United Kingdom	Europe	High-income economies	Practice nurse; specialist nurse; link worker; general practitioner	Enhanced care (package): Medication review or intensification; guideline-based structured care; more frequent follow-up	Intensive care	3 How care is delivered
6	(Berry, Williams et al. 2015)	Randomized controlled trial	United States	North America	High-income economies	Physician or nurse practitioner	Interdisciplinary approach: Multidisciplinary team care	Multidisciplinary	1 Who provides care
7	(Blackberry, Furler et al. 2013)	Cluster randomized controlled trial	Australia	Oceania	High-income economies	General practices and practice nurses	Telephone coaching: Telephone support	Education	2 What care is provided
8	(Bray, Cummings et al. 2013)	Quasi-experimental study	United States	North America	High-income economies	Nurse; pharmacist; and dietitian	Diabetes care management team: Care/case management; medication review or intensification	Multidisciplinary	1 Who provides care
9	(Brown, Garcia et al. 2002)	Randomized repeated-measures study	United States	North America	High-income economies	Nurses; dietitians; and community workers.	Diabetes self-management: Self-management support	Self-management	1 Who provides care
10	(Buhse, Kuniss et al. 2018)	two-arm, multicenter cluster RCT	Germany	Europe	High-income economies	Family physicians	Informed shared decision-making: Medication review or intensification	Shared decision making	3 How care is delivered
11	(Cabré Font, Colungo Francia et al. 2021)	Pre-post intervention study	Germany	Europe	High-income economies	Diabetes specialist nurse; endocrinology	Diabetes education program: Patient education	Education	2 What care is provided
12	(Carallo, Scavelli et al. 2015)	Non-randomized controlled trial	Italy	Europe	High-income economies	General practitioners; Hospital Consultants	Telemedicine (integrated care program): Web/mobile support; electronic records/decision support	Telemedicine	4 Information and communication technology
13	(Chaves-Fonseca, Matos et al. 2009)	Randomized controlled cohort study	Brazil	South America	Upper-middle-income economies	Doctors; nurses; pharmacists and health technicians	Staged diabetes management: Guideline-based structured care	Disease management	3 How care is delivered
14	(Chiu, Hu et al. 2016)	Randomized controlled trial	China	Asia	Upper-middle-income economies	Nurses; two assistants whose academic backgrounds were nursing and psychology	Minimal psychological intervention: Psychological support	Psychological	2 What care is provided
15	(Cho, Kwon et al. 2011)	Randomized controlled trial	South Korea	Asia	High-income economies	Nurse; physician; diabetes educator	Remote coaching system: Telemonitoring and feedback	Telemedicine	4 Information and communication technology

No.	Study	Study design	Country/region	Continent	Income level	Provider	Intervention content	Intervention subcategory	Intervention category
16	(Cleveringa, Gorter et al. 2007)	Before-and-after study	Netherlands	Europe	High-income economies	Practice nurse	Computerized decision support systems: Electronic records/decision support	The use of information and communication technology	4 Information and communication technology
17	(Dale, Caramlau et al. 2009)	Randomized controlled trial	United Kingdom	Europe	High-income economies	Peer supports; diabetes specialist nurses	Telephone support: Medication review or intensification	The use of information and communication technology	4 Information and communication technology
18	(De Greef, Deforche et al. 2011)	Randomized controlled trial	Belgium	Europe	High-income economies	General practitioners; behavior expert	Counseling: Behavior-change counseling	Interview	2 What care is provided
19	(De la Fuente Coria, Cruz-Cobo et al. 2020)	Randomized controlled trial	Spain	Europe	High-income economies	Nurse	Education program: Patient education	Education	2 What care is provided
20	(Denig, Schuling et al. 2014)	Randomized controlled trial	Netherlands	Europe	High-income economies	Health providers	Patient oriented decision aid: Shared decision-making	The use of information and communication technology	4 Information and communication technology
21	(DePue, Dunsiger et al. 2013)	Randomized study	United States	North America	High-income economies	A nurse case manager with four Community health workers	Nurse–community health worker team: Multidisciplinary team-based diabetes care	Multidisciplinary	1 Who provides care
22	(Dijkstra, Braspenning et al. 2008)	Randomized controlled trial	Netherlands	Europe	High-income economies	General practitioners	Diabetes passports: Technology-supported diabetes care	The use of information and communication technology	4 Information and communication technology
23	(du Pon, El Azzati et al. 2019)	Randomized controlled trial	Netherlands	Europe	High-income economies	General practitioners; Dietitian and a PN	Proactive interdisciplinary self-management: Multidisciplinary team care; provider training; self-management support	Multidisciplinary	1 Who provides care
24	(Edelman, Dolor et al. 2015)	Randomized controlled trial	United States	North America	High-income economies	Nurse	Nurse behavioral management: Behavior-change counseling	Behavior	2 What care is provided
25	(Edelman, Fredrickson et al. 2010)	Randomized controlled trial	United States	North America	High-income economies	Primary care general internist; a pharmacist; and a nurse or other certified diabetes educator.	Group medical clinics: Multidisciplinary team-based diabetes care	Multidisciplinary	1 Who provides care
26	(Farmer, Hardeman et al. 2012)	Randomized controlled trial	United Kingdom	Europe	High-income economies	Clinical nurse clinical psychologist and intervention facilitator	Consultation intervention: Patient counseling and communication support	Interview	2 What care is provided
27	(Fisher, Polonsky et al. 2011)	Cluster randomized controlled trial	United States	North America	High-income economies	Physicians	Self-monitoring of blood glucose: Self-management support	Self-management	1 Who provides care
28	(Frei, Senn et al. 2014)	Cluster randomized controlled trial	Switzerland	Europe	High-income economies	Primary care physicians and practice nurses	Trained practice nurse in implementation of the chronic care model: Provider training; guideline-based structured care	Disease management	3 How care is delivered
29	(Frosch, Uy et al. 2011)	Randomized controlled trial	United States	North America	High-income economies	Nurse	Behavior support intervention: Provider training; behavior-change counseling; telephone support	Behavior	2 What care is provided
30	(Furler, O'Neal et al. 2017)	Cluster randomized controlled trial	Australia	Oceania	High-income economies	Practice nurse; specialist registered nurse; General practitioners	Novel model of care (reorientation of the roles of health professionals): Medication review or intensification	Disease management	3 How care is delivered

No.	Study	Study design	Country/region	Continent	Income level	Provider	Intervention content	Intervention subcategory	Intervention category
31	(Gabbay, Añel-Tiangco et al. 2013)	Randomized controlled trial	United States	North America	High-income economies	Nurse care management	Addition of nurse case managers (ncms): Provider training; behavior-change counseling	Interview	2 What care is provided
32	(Gallegos, Ovalle-Berúmen et al. 2006)	Quasi-experimental study	Mexico	North America	Upper-middle-income economies	Nurse	Education and counseling: Behavior-change counseling	Combination	2 What care is provided
33	(Glasgow, Nutting et al. 2005)	Cluster randomized controlled trial	United States	North America	High-income economies	Physicians; care manager (usually a nurse or medical assistant	Computer assisted intervention: Technology-supported diabetes care	The use of information and communication technology	4 Information and communication technology
34	(Grant, Wald et al. 2008)	Randomized controlled trial	United States	North America	High-income economies	Primary care physicians	Online personal health records: Web/mobile support; electronic records/decision support	Health information systems	4 Information and communication technology
35	(Grillo Mde, Neumann et al. 2016)	Randomized controlled trial	Brazil	South America	Upper-middle-income economies	Nurse	Structured group education program: Patient education	Education	2 What care is provided
36	(Groeneveld, Petri et al. 2001)	Randomized controlled trial	Netherlands	Europe	High-income economies	General practitioners; diabetes educator (a nurse) and a Dietitian	Structured care assistance: Guideline-based structured care	Multidisciplinary	1 Who provides care
37	(Guldberg, Vedsted et al. 2011)	Cluster randomized controlled trial	Denmark	Europe	High-income economies	General practitioners	Electronic feedback system: Electronic records/decision support; reminders and feedback	Health information systems	4 Information and communication technology
38	(Hayashino, Suzuki et al. 2016)	Cluster randomized controlled trial	Japan	Asia	High-income economies	Physicians	Multifaceted interventions: Technology-supported diabetes care	Health information systems	4 Information and communication technology
39	(Hiss, Armbruster et al. 2007)	Randomized controlled trial	United States	North America	High-income economies	Nurse care manager	Nurse care manager: Care/case management	Multidisciplinary	1 Who provides care
40	(Holbrook, Thabane et al. 2009)	Randomized controlled trial	Canada	North America	High-income economies	Primary care providers	Individualized electronic decision support and reminders: Electronic records/decision support; reminders and feedback	The use of information and communication technology	4 Information and communication technology
41	(Houweling, Kleefstra et al. 2011)	Randomized controlled trial	Netherlands	Europe	High-income economies	Practice nurses	Practice nurses: Task shifting within the diabetes care team	Task shifting	1 Who provides care
42	(Ismail, Winkley et al. 2018)	Randomized controlled trial	United Kingdom	Europe	High-income economies	Practice nurses	Psychological intervention: Behavior-change counseling	Psychological	2 What care is provided
43	(Jansink, Braspenning et al. 2013)	Cluster randomized controlled trial	Netherlands	Europe	High-income economies	Nurse	Motivational interviewing (integrates patient centered lifestyle counselling): Behavior-change counselling	Interview	2 What care is provided
44	(Jayasuriya, Pinidiyapathirage et al. 2015)	Randomized controlled trial	Sri Lanka	Asia	Lower-middle-income economies	Nurses	Diabetes self-management: Provider training; self-management support	Self-management	1 Who provides care
45	(Jia, Zhang et al. 2021)	Cluster randomized controlled trial	China	Asia	Upper-middle-income economies	Primary care doctors; county doctors	Mobile health: Web/mobile support	The use of information and communication technology	4 Information and communication technology
46	(Jutterström, Hörnsten et al. 2016)	Randomized controlled trial	Sweden	Europe	High-income economies	Diabetes specialist nurses; nurses	Self-management support: Self-management support	Self-management	1 Who provides care

No.	Study	Study design	Country/region	Continent	Income level	Provider	Intervention content	Intervention subcategory	Intervention category
47	(Juul, Maindal et al. 2014)	Cluster randomized controlled trial	Denmark	Europe	High-income economies	General Practice Nurses	Training course: Provider training	Interview	2 What care is provided
48	(Khan, Walley et al. 2018)	Cluster randomized controlled trial	Pakistan	Asia	Lower-middle-income economies	Doctors; allied professionals	Case management: Care/case management	Case management	3 How care is delivered
49	(Kim, Kim et al. 2015)	Randomized controlled trial	United States	North America	High-income economies	Nurses, community health workers	Behavioral intervention program: Behavior-change counseling	Behavior	2 What care is provided
50	(Kinmonth, Woodcock et al. 1998)	Randomized controlled trial	United Kingdom	Europe	High-income economies	Doctors; nurses	Patient centred care: Provider training	Patient-centered care	2 What care is provided
51	(Krein, Klamerus et al. 2004)	Randomized controlled trial	United States	North America	High-income economies	Nurse practitioner case managers; primary care providers	Collaborative case management: Care/case management	Case management	3 How care is delivered
52	(Lamers, Jonkers et al. 2011)	Randomized controlled trial	Netherlands	Europe	High-income economies	Nurses	Psychological intervention: Self-management support	Psychological	2 What care is provided
53	(Lohmann, Siersma et al. 2010)	Non-randomized controlled trial	Denmark	Europe	High-income economies	General practitioners	Motivational interviewing combined with fitness tests: Behavior-change counseling; diet and physical-activity support	Interview	2 What care is provided
54	(Lou, Ye et al. 2020)	Randomized controlled trial	China	Asia	Upper-middle-income economies	General practitioners; nurses; An expert team （endocrinologists, diabetes specialists, and nurses）	Intensified diabetes management program: Self-management support; more frequent follow-up	Disease management	3 How care is delivered
55	(Luchsinger, Palmas et al. 2011)	Randomized controlled trial	United States	North America	High-income economies	Nurses case managers	Diabetes case management: Care/case management	Case management	3 How care is delivered
56	(Maddigan, Majumdar et al. 2004)	Before-and-after study	Canada	North America	High-income economies	Primary care providers; physicians and diabetes educators	Multifaceted provider-level intervention: Diet and physical-activity support	Multidisciplinary	1 Who provides care
57	(Maislos and Weisman 2004)	prospective, randomized study	Israel	Asia	High-income economies	Team diabetologist; the Dietitian and the diabetes nurse educator	Multidisciplinary approach: Multidisciplinary team care	Multidisciplinary	1 Who provides care
58	(Markle-Reid, Ploeg et al. 2018)	Randomized controlled trial	Canada	North America	High-income economies	Team: An interprofessional team of registered nurses (RNs) and registered dietitians (RDs) from DECs; a program coordinator (PC) from a community partner	Community program: Multidisciplinary team-based diabetes care	Multidisciplinary	1 Who provides care
59	(Martos-Cabrera, Gómez-Urquiza et al. 2021)	Quasi-experimental study	Spain	Europe	High-income economies	Nurses	Education intervention: Patient education	Education	2 What care is provided
60	(Mathers, Ng et al. 2012)	Cluster randomized controlled trial	United Kingdom	Europe	High-income economies	Doctors and/or the nurses	Patient decision aid: Provider training; shared decision-making	Shared decision making	3 How care is delivered
61	(Meulepas, Braspenning et al. 2007)	Non-randomized controlled trial	Netherlands	Europe	High-income economies	General practitioners	Diabetes support service: Technology-supported diabetes care	The use of information and communication technology	4 Information and communication technology

No.	Study	Study design	Country/region	Continent	Income level	Provider	Intervention content	Intervention subcategory	Intervention category
62	(Meulepas, Braspenning et al. 2008)	Before-and-after study	Netherlands	Europe	High-income economies	Practice nurse	Patient-oriented interventions: Patient-centered diabetes care	Patient-centered care	2 What care is provided
63	(Moinfar, Sedaghat et al. 2016)	Before-and-after study	Iran	Asia	Upper-middle-income economies	General practitioners / primary care teams [A general practitioner; a nurse; and a nutritionist]	Collaborative care program (mental disorders among diabetic patients): Care/case management	Multidisciplinary	1 Who provides care
64	(Mons, Raum et al. 2013)	Randomized controlled trial	Germany	Europe	High-income economies	Practice nurses	Supportive counseling intervention: Behavior-change counseling; telephone support; more frequent follow-up	Interview	2 What care is provided
65	(Moreo, Sapir et al. 2015)	Before-and-after study	United States	North America	High-income economies	Primary care physicians	Quality improvement program to physicians (paced education intervention): Reminders and feedback	Education	2 What care is provided
66	(Musacchio, Lovagnini Scher et al. 2011)	Before-and-after study	Italy	Europe	High-income economies	Three diabetologists; two nurses and one dietitian	Chronic care model based on patient empowerment: Guideline-based structured care	Disease management	3 How care is delivered
67	(Najar, Tehrani et al. 2017)	Randomized controlled trial	Iran	Asia	Upper-middle-income economies	Physicians communication network: a family physician; an internist; and an endocrinologist; endocrinologist：	Developing a communication network among physicians: Multidisciplinary team-based diabetes care	Multidisciplinary	1 Who provides care
68	(Navicharern, Aungsuroch et al. 2009)	Quasi-experimental study	Thailand	Asia	Upper-middle-income economies	Nurses	Multifaceted nurse-coaching intervention: Multicomponent diabetes care intervention	Multifaceted	5 Multifaceted
69	(Nesari, Zakerimoghadam et al. 2010)	Randomized study	Iran	Asia	Upper-middle-income economies	Nurse	Telephone follow-up: Telephone support	The use of information and communication technology	4 Information and communication technology
70	(Nishijima, Sarti et al. 2019)	Quasi-experimental study	China	Asia	Upper-middle-income economies	Nurse	Adherence to a diabetes therapeutic regimen: Multidisciplinary team care	Multidisciplinary	1 Who provides care
71	(Noda, Hayashino et al. 2020)	Cluster randomized controlled trial	Japan	Asia	High-income economies	Primary care providers	Triple-faceted intervention: Reminders and feedback	Multifaceted	5 Multifaceted
72	(O'Connor, Sperl-Hillen et al. 2011)	Cluster randomized controlled trial	United States	North America	High-income economies	Primary care physicians; nurses	Clinical decision support: Electronic records/decision support	The use of information and communication technology	4 Information and communication technology
73	(Odnoletkova, Goderis et al. 2016)	Randomized controlled trial	Belgium	Europe	High-income economies	Nurse educator	Telecoaching intervention: Telephone support	Education	2 What care is provided
74	(Olivarius, Beck-Nielsen et al. 2001)	Randomized controlled trial	Denmark	Europe	High-income economies	General practitioners	Multifaceted intervention directed at general practitioners: Guideline-based structured care	Multifaceted	5 Multifaceted
75	(Olry de Labry Lima, Bermúdez Tamayo et al. 2017)	Cluster randomized controlled trial	Spain	Europe	High-income economies	Physicians	Patient-practitioner communication intervention: Self-management support	Self-management	1 Who provides care
76	(Panisch, Johansson et al. 2018)	Cluster randomized controlled trial	Austria	Europe	High-income economies	Family physicians	Disease management program: Guideline-based structured care	Disease management	3 How care is delivered
77	(Peterson, Radosevich et al. 2008)	Randomized controlled trial	United States	North America	High-income economies	Physicians	Multicomponent organizational intervention: Electronic records/decision support; reminders and feedback	Multifaceted	5 Multifaceted

No.	Study	Study design	Country/region	Continent	Income level	Provider	Intervention content	Intervention subcategory	Intervention category
78	(Piette, Richardson et al. 2011)	Randomized controlled trial	United States	North America	High-income economies	Nurses	Telephone-delivered cognitive behavioral therapy: Behavior-change counseling; telephone support	Behavior	2 What care is provided
79	(Pill, Stott et al. 1998)	Randomized controlled trial	United Kingdom	Europe	High-income economies	General practitioners; research nurse; psychologist	Training in a patient-centered intervention for physicians: Provider training	Patient-centered care	2 What care is provided
80	(Polonsky, Fisher et al. 2011)	Cluster randomized controlled trial	United States	North America	High-income economies	Physicians	Structured self-monitoring of blood glucose (smbg): Self-management support	Self-management	1 Who provides care
81	(Powers, Grubber et al. 2012)	Randomized controlled trial	United Kingdom	Europe	High-income economies	Nurse	Telephone intervention: Self-management support; medication review or intensification; telephone support; more frequent follow-up	The use of information and communication technology	4 Information and communication technology
82	(Prestes, Gayarre et al. 2017)	random	Argentina	South America	Upper-middle-income economies	Physicians; nurses	Multistrategic approach: Multicomponent diabetes care intervention	Multifaceted	5 Multifaceted
83	(Ramallo-Fariña, García-Bello et al. 2020)	Randomized controlled trial	Spain	Europe	High-income economies	Physicians and nurses	Internet-based multicomponent interventions: Web/mobile support	The use of information and communication technology	4 Information and communication technology
84	(Reutens, Hutchinson et al. 2012)	Randomized controlled trial	China; South Korea; Vietnam; Philippines; Thailand; Indonesia; Singapore; Malaysia	Asia	Mixed: high- and upper-middle-income economies	General practitioners	Education of doctors about guidelines: Provider training; guideline-based structured care	Education	2 What care is provided
85	(Ridgeway, Harvill et al. 1999)	Randomized controlled trial	United States	North America	High-income economies	Nurse; dietitian; physicians	Education and behavior modification classes: Behavior-change counseling	Combination	2 What care is provided
86	(Rubak, Sandbaek et al. 2009)	Randomized controlled trial	Denmark	Europe	High-income economies	General practitioners	Motivational interview for GPs: Provider training; behavior-change counseling	Interview	2 What care is provided
87	(Sönnichsen, Winkler et al. 2010)	Cluster randomized controlled trial	Austria	Europe	High-income economies	General practitioners internists	Disease management program: Guideline-based structured care	Disease management	3 How care is delivered
88	(Sperl-Hillen, O'Connor et al. 2010)	Cluster randomized controlled trial	United States	North America	High-income economies	Primary care physicians (Primary care physicians	Simulated physician learning program: Provider training	The use of information and communication technology	4 Information and communication technology
89	(Stone, Rao et al. 2010)	Randomized controlled trial	United States	North America	High-income economies	Nurse	Active care management supported by home telemonitoring: Care/case management; telemonitoring and feedback	Telemedicine	4 Information and communication technology
90	(Sturt, Whitlock et al. 2008)	Cluster randomized controlled trial	United Kingdom	Europe	High-income economies	Nurses	Diabetes manual: Patient education; self-management support	Education	2 What care is provided
91	(Talavera, Castañeda et al. 2021)	Randomized controlled trial	United States	North America	High-income economies	Primary care providers and healthcare organizations; physician/mid-level medical provider and specialty behavioral health provider	Integrated team-based care intervention: Multidisciplinary team care	Multidisciplinary	1 Who provides care

No.	Study	Study design	Country/region	Continent	Income level	Provider	Intervention content	Intervention subcategory	Intervention category
92	(Tang, Overhage et al. 2013)	Randomized controlled trial	United States	North America	High-income economies	Nurse care managers and registered Dietitian (RD	Online disease management: Web/mobile support; guideline-based structured care	Telemedicine	4 Information and communication technology
93	(Taylor, Oberle et al. 2005)	Randomized study	Canada	North America	High-income economies	Nurse exercise specialist and/or nutritionist.	Nurse-physician collaborative approach: Multidisciplinary team care	Multidisciplinary	1 Who provides care
94	(Tourkmani, Abdelhay et al. 2018)	Randomized controlled trial	Saudi Arabia	Asia	High-income economies	Multidisciplinary program: a senior family physician; clinical pharmacy specialist who acted as a case manager; Dietitian; diabetic educator; health educator; and social worker.	Multidisciplinary program: Multidisciplinary team care	Multidisciplinary	1 Who provides care
95	(van Bruggen, Gorter et al. 2008)	Cluster randomized controlled trial	Netherlands	Europe	High-income economies	Nurses led; four General practitioners; two internists from the local hospital; three diabetes nurse specialists and two Dietitians	Multifaceted intervention to implement a locally adapted guideline: Guideline-based structured care	Multifaceted	5 Multifaceted
96	(van Dijk-de Vries, van Bokhoven et al. 2015)	Cluster randomized controlled trial	Netherlands	Europe	High-income economies	Practice nurses	Self-management support: Diabetes self-management support	Self-management	1 Who provides care
97	(Varroud-Vial, Simon et al. 2004)	Randomized study	France	Europe	High-income economies	General practitioners	Staged diabetes management: Guideline-based structured care	Disease management	3 How care is delivered
98	(Wakefield, Holman et al. 2011)	Randomized controlled trial	United States	North America	High-income economies	Nurse team (nurses; a physician; and a certified diabetes educator	Home telehealth intervention: Technology-supported diabetes care	Telemedicine	4 Information and communication technology
99	(Weinberger, Kirkman et al. 1995)	Randomized controlled trial	United States	North America	High-income economies	Nurses	Telephone based education: Telephone support; more frequent follow-up	Education	2 What care is provided
100	(Weitzman, Greenfield et al. 2009)	Cluster randomized controlled trial	Israel	Asia	High-income economies	Primary care practitioner	Adding a simple patient intervention to physician feedback: Telephone support; reminders and feedback	Patient reminder	3 How care is delivered
101	(Welch, Allen et al. 2011)	Randomized controlled trial	United States	North America	High-income economies	Nurses led and educator	Diabetes care program: Web/mobile support; guideline-based structured care	The use of information and communication technology	4 Information and communication technology
102	(Welch, Zagarins et al. 2015)	Randomized controlled trial	United States	North America	High-income economies	A diabetes nurse led or diabetes dietitian(educator) ; Diabetes nurses; diabetes dietitians; diabetes educator	Internet-based diabetes management platform: Web/mobile support	Telemedicine	4 Information and communication technology
103	(Whitehead, Crowe et al. 2017)	Randomized controlled trial	New Zealand	Oceania	High-income economies	Primary care nurses	Education and cognitive behavior therapy: Behavior-change counseling	Combination	2 What care is provided
104	(Wild, Hanley et al. 2016)	Randomized controlled trial	United Kingdom	Europe	High-income economies	Nurses; family practice clinicians	Supported telemonitoring: Telemonitoring and feedback	Telemedicine	4 Information and communication technology
105	(Wollny, Altiner et al. 2019)	Cluster randomized controlled trial	Germany	Europe	High-income economies	General practitioners	Shared decision making: Shared decision-making	Shared decision making	3 How care is delivered
106	(Woodard, Amspoker et al. 2022)	Randomized controlled trial	United States	North America	High-income economies	Physicians; nurse educators; nurse practitioners; pharmacists; dietitians; and psychologists	Collaborative goal setting with enhanced education: Shared decision-making	Education	2 What care is provided
107	(Xu, Dong et al. 2021)	Cluster randomized controlled trial	China	Asia	Upper-middle-income economies	General practitioners	Group cognitive behavioral therapy: Behavior-change counseling	Behavior	2 What care is provided

No.	Study	Study design	Country/region	Continent	Income level	Provider	Intervention content	Intervention subcategory	Intervention category
108	(Yang, Lee et al. 2020)	Cluster randomized controlled trial	South Korea	Asia	High-income economies	Physicians	Mobile phone–based glucose-monitoring and feedback system: Web/mobile support; telemonitoring and feedback; reminders and feedback	Telemedicine	4 Information and communication technology
109	(Young, Miyamoto et al. 2020)	Randomized controlled trial	United States	North America	High-income economies	Nurses	Nurse coaching using motivational interviewing paired with mobile health: Self-management support; behavior-change counseling; web/mobile support	Interview	2 What care is provided
110	(Young, Taylor et al. 2005)	Randomized controlled trial	United Kingdom	Europe	High-income economies	Diabetes specialist nurse	Pro-active call center treatment support: Telephone support; guideline-based structured care	The use of information and communication technology	4 Information and communication technology
111	(Zandiyeh, Hedayati et al. 2015)	Before-and-after study	Iran	Asia	Upper-middle-income economies	Nurses	Educational intervention: Structured diabetes education	Education	2 What care is provided
112	(Zhang, Zhang et al. 2021)	Cluster randomized controlled trial	China	Asia	Upper-middle-income economies	General practitioners	Cognitive behavior therapy: Behavior-change counseling	Behavior	2 What care is provided

Reference

Akturan, S., Ç. Kaya, P. C. Ünalan and M. Akman (2017). "The effect of the BATHE interview technique on the empowerment of diabetic patients in primary care: a cluster randomised controlled study." Primary care diabetes **11**(2): 154-161.

Al Asmary, S. M., T. Al-Harbi, A. M. Tourkmani, H. I. Al Khashan, H. Al-Qahtani, A. Mishriky, A. Bakhiet and N. A. Al Nowaiser (2013). "Impact of integrated care program on glycemic control and cardiovascular risk in adult patients with type 2 diabetes." Journal of Clinical Outcomes Management **20**(8): 356-363.

Allerton, J. and R. Mash (2020). "The impact of intensified clinical care on glycaemic control in patients with type 2 diabetes at Khayelitsha Community Health Centre, South Africa: Quasi-experimental study." Prim Care Diabetes **14**(2): 97-103.

Alonso-Domínguez, R., M. C. Patino-Alonso, N. Sánchez-Aguadero, L. García-Ortiz, J. I. Recio-Rodríguez and M. A. Gómez-Marcos (2019). "Effect of a multifactorial intervention on the increase in physical activity in subjects with type 2 diabetes mellitus: a randomized clinical trial (EMID Study)." Eur J Cardiovasc Nurs **18**(5): 399-409.

Bellary, S., J. O'Hare, N. Raymond, A. Gumber, S. Mughal, A. Szczepura, S. Kumar and A. Barnett (2008). "Enhanced diabetes care to patients of south Asian ethnic origin (the United Kingdom Asian Diabetes Study): a cluster randomised controlled trial." The Lancet **371**(9626): 1769-1776.

Berry, D. C., W. Williams, E. G. Hall, R. Heroux and T. Bennett-Lewis (2015). "Imbedding Interdisciplinary Diabetes Group Visits Into a Community-Based Medical Setting." Diabetes Educ **42**(1): 96-107.

Blackberry, I. D., J. S. Furler, J. D. Best, P. Chondros, M. Vale, C. Walker, T. Dunning, L. Segal, J. Dunbar, R. Audehm and et al. (2013). "Effectiveness of general practice based, practice nurse led telephone coaching on glycaemic control of type 2 diabetes: the Patient Engagement and Coaching for Health (PEACH) pragmatic cluster randomised controlled trial." BMJ (Clinical research ed.) **347**: f5272.

Bray, P., D. M. Cummings, S. Morrissey, D. Thompson, D. Holbert, K. Wilson, E. Lukosius and R. Tanenberg (2013). "Improved outcomes in diabetes care for rural African Americans." Ann Fam Med **11**(2): 145-150.

Brown, S. A., A. A. Garcia, K. Kouzekanani and C. L. Hanis (2002). "Culturally competent diabetes self-management education for Mexican Americans: the Starr County border health initiative." Diabetes Care **25**(2): 259-268.

Buhse, S., N. Kuniss, K. Liethmann, U. A. Müller, T. Lehmann and I. Mühlhauser (2018). "Informed shared decision-making programme for patients with type 2 diabetes in primary care: cluster randomised controlled trial." BMJ Open **8**(12): e024004.

Cabré Font, C., C. Colungo Francia, I. Vinagre Torres, M. Jansà i Morató and I. Conget Donlo (2021). "A Therapeutic Education Program with a Diabetes Specialist Nurse for Type 2 Diabetes Patients Using Insulin in a Primary Care Setting. A Diabetes Education Program with a Diabetes Specialist Nurse in a Primary Care Setting." Endocrinologia, Diabetes y Nutricion **68**(9): 628-635.

Carallo, C., F. B. Scavelli, M. Cipolla, V. Merante, V. Medaglia, C. Irace and A. Gnasso (2015). "Management of type 2 diabetes mellitus through telemedicine." PLoS ONE **10**(5).

Chaves-Fonseca, R. M., O. S. Matos, R. A. Lordelo, M. Abreu, M. G. Farias, J. F. Coutinho, M. N. Ribeiro, L. Matteoni-Athayde, I. Lessa, J. Pousada, M. Oliveira, C. Lopes, E. Strock and R. Mazze (2009). "Implementation of a systematic approach to diabetes in primary care in Bahia, Brazil improves metabolic outcomes: PRODIBA-Programa de Interiorização da Assistência ao Diabetes na Bahia (Project for Dissemination of Diabetes Care in the State of Bahia)." Diabet Med **26**(3): 286-292.

Chiu, C. J., Y. H. Hu, L. A. Wray, E. A. Beverly, Y. C. Yang, J. S. Wu and F. H. Lu (2016). "Dissemination of evidence-base minimal psychological intervention for diabetes management in Taiwan adults with type 2 diabetes." International Journal of Clinical and Experimental Medicine **9**(7): 14489-14498.

Cho, J.-H., H.-S. Kwon, H.-S. Kim, J.-A. Oh and K.-H. Yoon (2011). "Effects on diabetes management of a health-care provider mediated, remote coaching system via a PDA-type glucometer and the Internet." Journal of Telemedicine & Telecare **17**(7): 365-370.

Cleveringa, F. G., K. J. Gorter, M. van den Donk, P. L. Pijman and G. E. Rutten (2007). "Task delegation and computerized decision support reduce coronary heart disease risk factors in type 2 diabetes patients in primary care." Diabetes Technol Ther **9**(5): 473-481.
Dale, J., I. Caramlau, J. Sturt, T. Friede and R. Walker (2009). "Telephone peer-delivered intervention for diabetes motivation and support: the telecare exploratory RCT." Patient Educ Couns **75**(1): 91-98.
De Greef, K., B. Deforche, C. Tudor-Locke and I. De Bourdeaudhuij (2011). "Increasing physical activity in Belgian type 2 diabetes patients: a three-arm randomized controlled trial." Int J Behav Med **18**(3): 188-198.
De la Fuente Coria, M. C., C. Cruz-Cobo and M. J. Santi-Cano (2020). "Effectiveness of a primary care nurse delivered educational intervention for patients with type 2 diabetes mellitus in promoting metabolic control and compliance with long-term therapeutic targets: Randomised controlled trial." Int J Nurs Stud **101**: 103417.
Denig, P., J. Schuling, F. Haaijer-Ruskamp and J. Voorham (2014). "Effects of a patient oriented decision aid for prioritising treatment goals in diabetes: pragmatic randomised controlled trial." Bmj **349**: g5651.
DePue, J. D., S. Dunsiger, A. D. Seiden, J. Blume, R. K. Rosen, M. G. Goldstein, O. Nu'usolia, J. Tuitele and S. T. McGarvey (2013). "Nurse-community health worker team improves diabetes care in American Samoa: results of a randomized controlled trial." Diabetes Care **36**(7): 1947-1953.
Dijkstra, R., J. Braspenning and R. Grol (2008). "Implementing diabetes passports to focus practice reorganization on improving diabetes care." Int J Qual Health Care **20**(1): 72-77.
du Pon, E., S. El Azzati, A. van Dooren, N. Kleefstra, E. Heerdink and S. van Dulmen (2019). "Effects of a Proactive Interdisciplinary Self-Management (PRISMA) program on medication adherence in patients with type 2 diabetes in primary care: a randomized controlled trial." Patient Prefer Adherence **13**: 749-759.
Edelman, D., R. J. Dolor, C. J. Coffman, K. C. Pereira, B. B. Granger, J. H. Lindquist, A. M. Neary, A. J. Harris and H. B. Bosworth (2015). "Nurse-led behavioral management of diabetes and hypertension in community practices: a randomized trial." J Gen Intern Med **30**(5): 626-633.
Edelman, D., S. K. Fredrickson, S. D. Melnyk, C. J. Coffman, A. S. Jeffreys, S. Datta, G. L. Jackson, A. C. Harris, N. S. Hamilton, H. Stewart, J. Stein and M. Weinberger (2010). "Medical clinics versus usual care for patients with both diabetes and hypertension: a randomized trial." Ann Intern Med **152**(11): 689-696.
Farmer, A., W. Hardeman, D. Hughes, A. T. Prevost, Y. Kim, A. Craven, J. Oke, S. Boase, M. Selwood, I. Kellar and et al. (2012). "An explanatory randomised controlled trial of a nurse-led, consultation-based intervention to support patients with adherence to taking glucose lowering medication for type 2 diabetes." BMC family practice **13**: 30.
Fisher, L., W. Polonsky, C. G. Parkin, Z. Jelsovsky, L. Amstutz and R. S. Wagner (2011). "The impact of blood glucose monitoring on depression and distress in insulin-naïve patients with type 2 diabetes." Curr Med Res Opin **27 Suppl 3**: 39-46.
Frei, A., O. Senn, C. Chmiel, J. Reissner, U. Held and T. Rosemann (2014). "Implementation of the chronic care model in small medical practices improves cardiovascular risk but not glycemic control." Diabetes Care **37**(4): 1039-1047.
Frosch, D. L., V. Uy, S. Ochoa and C. M. Mangione (2011). "Evaluation of a behavior support intervention for patients with poorly controlled diabetes." Archives of internal medicine **171**(22): 2011-2017.
Furler, J., D. O'Neal, J. Speight, J. A. Manski-Nankervis, A. Gorelik, E. Holmes-Truscott, L. Ginnivan, D. Young, J. Best, dean and et al. (2017). "Supporting insulin initiation in type 2 diabetes in primary care: results of the Stepping Up pragmatic cluster randomised controlled clinical trial." BMJ (Clinical research ed.) **356**: j783.
Gabbay, R. A., R. M. Añel-Tiangco, C. Dellasega, D. T. Mauger, A. Adelman and D. H. Van Horn (2013). "Diabetes nurse case management and motivational interviewing for change (DYNAMIC): results of a 2-year randomized controlled pragmatic trial." J Diabetes **5**(3): 349-357.
Gallegos, E. C., F. Ovalle-Berúmen and M. V. Gomez-Meza (2006). "Metabolic control of adults with type 2 diabetes mellitus through education and counseling." Journal of Nursing Scholarship **38**(4): 344-351.
Glasgow, R. E., P. A. Nutting, D. K. King, C. C. Nelson, G. Cutter, B. Gaglio, A. K. Rahm and H. Whitesides (2005). "Randomized effectiveness trial of a computer-assisted intervention to improve diabetes care." Diabetes Care **28**(1): 33-39.

Grant, R. W., J. S. Wald, J. L. Schnipper, T. K. Gandhi, E. G. Poon, E. J. Orav, D. H. Williams, L. A. Volk and B. Middleton (2008). "Practice-linked online personal health records for type 2 diabetes mellitus: a randomized controlled trial." Arch Intern Med **168**(16): 1776-1782.
Grillo Mde, F., C. R. Neumann, S. F. Scain, R. F. Rozeno, L. Beloli, T. Perinetto, J. L. Gross and C. B. Leitão (2016). "Diabetes education in primary care: a randomized clinical trial." Cad Saude Publica **32**(5).
Groeneveld, Y., H. Petri, J. Hermans and M. Springer (2001). "An assessment of structured care assistance in the management of patients with type 2 diabetes in general practice." Scand J Prim Health Care **19**(1): 25-30.
Guldberg, T. L., P. Vedsted, J. K. Kristensen and T. Lauritzen (2011). "Improved quality of Type 2 diabetes care following electronic feedback of treatment status to general practitioners: a cluster randomized controlled trial." Diabet Med **28**(3): 325-332.
Hayashino, Y., H. Suzuki, K. Yamazaki, A. Goto, K. Izumi and M. Noda (2016). "A cluster randomized trial on the effect of a multifaceted intervention improved the technical quality of diabetes care by primary care physicians: The Japan Diabetes Outcome Intervention Trial-2 (J-DOIT2)." Diabet Med **33**(5): 599-608.
Hiss, R. G., B. A. Armbruster, M. L. Gillard and L. A. McClure (2007). "Nurse care manager collaboration with community-based physicians providing diabetes care: a randomized controlled trial." Diabetes Educ **33**(3): 493-502.
Holbrook, A., L. Thabane, K. Keshavjee, L. Dolovich, B. Bernstein, D. Chan, S. Troyan, G. Foster and H. Gerstein (2009). "Individualized electronic decision support and reminders to improve diabetes care in the community: COMPETE II randomized trial." Cmaj **181**(1-2): 37-44.
Houweling, S. T., N. Kleefstra, K. J. van Hateren, K. H. Groenier, B. Meyboom-de Jong and H. J. Bilo (2011). "Can diabetes management be safely transferred to practice nurses in a primary care setting? A randomised controlled trial." J Clin Nurs **20**(9-10): 1264-1272.
Ismail, K., K. Winkley, N. de Zoysa, A. Patel, M. Heslin, H. Graves, S. Thomas, D. Stringer, D. Stahl and S. A. Amiel (2018). "Nurse-led psychological intervention for type 2 diabetes: a cluster randomised controlled trial (Diabetes-6 study) in primary care." Br J Gen Pract **68**(673): e531-e540.
Jansink, R., J. Braspenning, E. Keizer, T. van der Weijden, G. Elwyn and R. Grol (2013). "No identifiable Hb1Ac or lifestyle change after a comprehensive diabetes programme including motivational interviewing: a cluster randomised trial." Scand J Prim Health Care **31**(2): 119-127.
Jayasuriya, R., M. J. Pinidiyapathirage, R. Jayawardena, A. Kasturiratne, P. de Zoysa, P. Godamunne, S. Gamage and A. R. Wickremasinghe (2015). "Translational research for Diabetes Self-Management in Sri Lanka: a randomized controlled trial." Primary care diabetes **9**(5): 338-345.
Jia, W., P. Zhang, D. Zhu, N. Duolikun, H. Li, Y. Bao and X. Li (2021). "Evaluation of an mHealth-enabled hierarchical diabetes management intervention in primary care in China (ROADMAP): A cluster randomized trial." PLoS Med **18**(9): e1003754.
Jutterström, L., Å. Hörnsten, H. Sandström, H. Stenlund and U. Isaksson (2016). "Nurse-led patient-centered self-management support improves HbA1c in patients with type 2 diabetes—A randomized study." Patient Education and Counseling **99**(11): 1821-1829.
Juul, L., H. T. Maindal, V. Zoffmann, M. Frydenberg and A. Sandbaek (2014). "Effectiveness of a training course for general practice nurses in motivation support in type 2 diabetes care: a cluster-randomised trial." PLoS One **9**(5): e96683.
Khan, M. A., J. D. Walley, N. Khan, J. Hicks, M. Ahmed, S. E. Khan, M. A. Khan, H. J. Khan and A. D. Harries (2018). "Effectiveness of an integrated diabetes care package at primary healthcare facilities: A cluster randomised trial in Pakistan." BJGP Open **2**(4): 1-11.
Kim, M. T., K. B. Kim, B. Huh, T. Nguyen, H.-R. Han, L. R. Bone and D. Levine (2015). "The Effect of a Community-Based Self-Help Intervention: Korean Americans With Type 2 Diabetes." American Journal of Preventive Medicine **49**(5): 726-737.
Kinmonth, A. L., A. Woodcock, S. Griffin, N. Spiegal and M. J. Campbell (1998). "Randomised controlled trial of patient centred care of diabetes in general practice: impact on current wellbeing and future disease risk. The Diabetes Care From Diagnosis Research Team." Bmj **317**(7167): 1202-1208.

Krein, S. L., M. L. Klamerus, S. Vijan, J. L. Lee, J. T. Fitzgerald, A. Pawlow, P. Reeves and R. A. Hayward (2004). "Case management for patients with poorly controlled diabetes: a randomized trial." Am J Med **116**(11): 732-739.
Lamers, F., C. C. Jonkers, H. Bosma, J. A. Knottnerus and J. T. van Eijk (2011). "Treating depression in diabetes patients: does a nurse-administered minimal psychological intervention affect diabetes-specific quality of life and glycaemic control? A randomized controlled trial." J Adv Nurs **67**(4): 788-799.
Lohmann, H., V. Siersma and N. F. Olivarius (2010). "Fitness consultations in routine care of patients with type 2 diabetes in general practice: an 18-month non-randomised intervention study." BMC Fam Pract **11**: 83.
Lou, Q., Q. Ye, H. Wu, Z. Wang, R. S. Ware, Y. Xiong and F. Xu (2020). "Effectiveness of a clinic-based randomized controlled intervention for type 2 diabetes management: an innovative model of intensified diabetes management in Mainland China (C-IDM study)." BMJ Open Diabetes Res Care **8**(1).
Luchsinger, J. A., W. Palmas, J. A. Teresi, S. Silver, J. Kong, J. P. Eimicke, R. S. Weinstock and S. Shea (2011). "Improved diabetes control in the elderly delays global cognitive decline." J Nutr Health Aging **15**(6): 445-449.
Maddigan, S. L., S. R. Majumdar, L. M. Guirguis, R. Z. Lewanczuk, T. K. Lee, E. L. Toth and J. A. Johnson (2004). "Improvements in patient-reported outcomes associated with an intervention to enhance quality of care for rural patients with type 2 diabetes: results of a controlled trial." Diabetes Care **27**(6): 1306-1312.
Maislos, M. and D. Weisman (2004). "Multidisciplinary approach to patients with poorly controlled type 2 diabetes mellitus: a prospective, randomized study." Acta Diabetol **41**(2): 44-48.
Markle-Reid, M., J. Ploeg, K. D. Fraser, K. A. Fisher, A. Bartholomew, L. E. Griffith, J. Miklavcic, A. Gafni, L. Thabane and R. Upshur (2018). "Community Program Improves Quality of Life and Self-Management in Older Adults with Diabetes Mellitus and Comorbidity." J Am Geriatr Soc **66**(2): 263-273.
Martos-Cabrera, M. B., J. L. Gómez-Urquiza, G. Cañadas-González, J. L. Romero-Bejar, N. Suleiman-Martos, G. A. Cañadas-De la Fuente and L. Albendín-García (2021). "Nursing-Intense Health Education Intervention for Persons with Type 2 Diabetes: A Quasi-Experimental Study." Healthcare (Basel) **9**(7).
Mathers, N., C. J. Ng, M. J. Campbell, B. Colwell, I. Brown and A. Bradley (2012). "Clinical effectiveness of a patient decision aid to improve decision quality and glycaemic control in people with diabetes making treatment choices: a cluster randomised controlled trial (PANDAs) in general practice." BMJ Open **2**(6).
Meulepas, M. A., J. C. Braspenning, W. J. de Grauw, A. E. Lucas, L. Harms, R. P. Akkermans and R. P. Grol (2007). "Logistic support service improves processes and outcomes of diabetes care in general practice." Fam Pract **24**(1): 20-25.
Meulepas, M. A., J. C. Braspenning, W. J. de Grauw, A. E. Lucas, D. Wijkel and R. P. Grol (2008). "Patient-oriented intervention in addition to centrally organised checkups improves diabetic patient outcome in primary care." Qual Saf Health Care **17**(5): 324-328.
Moinfar, Z., M. Sedaghat, F. Abolhassani, V. Sharifi, Z. Moinfar, S. Eftekhari and F. Mirzaaghaee (2016). "A collaborative care program for management of common mental disorders among diabetic patients in a primary healthcare setting." Journal of Public Health (Germany) **24**(4): 273-279.
Mons, U., E. Raum, H. U. Krämer, G. Rüter, D. Rothenbacher, T. Rosemann, J. Szecsenyi and H. Brenner (2013). "Effectiveness of a supportive telephone counseling intervention in type 2 diabetes patients: randomized controlled study." PLoS One **8**(10): e77954.
Moreo, K., T. Sapir and L. Greene (2015). "Applying Quality Improvement into Systems-based Learning to Improve Diabetes Outcomes in Primary Care." BMJ Qual Improv Rep **4**(1).
Musacchio, N., A. Lovagnini Scher, A. Giancaterini, L. Pessina, G. Salis, F. Schivalocchi, A. Nicolucci, F. Pellegrini and M. C. E. Rossi (2011). "Impact of a chronic care model based on patient empowerment on the management of Type2 diabetes: Effects of the SINERGIA programme." Diabetic Medicine **28**(6): 724-730.
Najar, A. V., H. Tehrani, H. Imamian, H. M. Hakak and M. Vahedian-Shahroodi (2017). "Impact of establishing a communication network of family physicians on level of Hba1cand FBS in patients with diabetes: A randomized clinical trial." Iranian Red Crescent Medical Journal **19**(11).
Navicharern, R., Y. Aungsuroch and S. Thanasilp (2009). "Effects of multifaceted nurse-coaching intervention on diabetic complications and satisfaction of persons with type 2 diabetes." J Med Assoc Thai **92**(8): 1102-1112.

Nesari, M., M. Zakerimoghadam, A. Rajab, S. Bassampour and S. Faghihzadeh (2010). "Effect of telephone follow-up on adherence to a diabetes therapeutic regimen." Jpn J Nurs Sci **7**(2): 121-128.
Nishijima, M., F. M. Sarti, I. Vodenska and G. Zhang (2019). "Effects of decentralization of primary health care on diabetes mellitus in Brazil." Public Health **166**: 108-120.
Noda, M., Y. Hayashino, K. Yamazaki, H. Suzuki, A. Goto, M. Kato, K. Izumi and M. Kobayashi (2020). "A cluster-randomized trial of the effectiveness of a triple-faceted intervention promoting adherence to primary care physician visits by diabetes patients." Sci Rep **10**(1): 2842.
O'Connor, P. J., J. M. Sperl-Hillen, W. A. Rush, P. E. Johnson, G. H. Amundson, S. E. Asche, H. L. Ekstrom and T. P. Gilmer (2011). "Impact of electronic health record clinical decision support on diabetes care: a randomized trial." Ann Fam Med **9**(1): 12-21.
Odnoletkova, I., G. Goderis, F. Nobels, S. Fieuws, B. Aertgeerts, L. Annemans and D. Ramaekers (2016). "Optimizing diabetes control in people with Type 2 diabetes through nurse-led telecoaching." Diabetic Medicine **33**(6): 777-785.
Olivarius, N. D. F., H. Beck-Nielsen, A. H. Andreasen, M. Hørder and P. A. Pedersen (2001). "Randomised controlled trial of structured personal care of type 2 diabetes mellitus." British Medical Journal **323**(7319): 970-975.
Olry de Labry Lima, A., C. Bermúdez Tamayo, G. Pastor Moreno, J. Bolívar Muñoz, I. Ruiz Pérez, M. Johri, F. Quesada Jiménez, P. Cruz Vela, A. M. de Los Ríos Álvarez, M. Prados Quel and et al. (2017). "Effectiveness of an intervention to improve diabetes self-management on clinical outcomes in patients with low educational level." Gaceta sanitaria **31**(1): 40-47.
Panisch, S., T. Johansson, M. Flamm, H. Winkler, R. Weitgasser and A. C. Sönnichsen (2018). "The impact of a disease management programme for type 2 diabetes on health-related quality of life: multilevel analysis of a cluster-randomised controlled trial." Diabetol Metab Syndr **10**: 28.
Peterson, K. A., D. M. Radosevich, P. J. O'Connor, J. A. Nyman, R. J. Prineas, S. A. Smith, T. J. Arneson, V. A. Corbett, J. C. Weinhandl, C. J. Lange and et al. (2008). "Improving Diabetes Care in Practice: findings from the TRANSLATE trial." Diabetes care **31**(12): 2238-2243.
Piette, J. D., C. Richardson, J. Himle, S. Duffy, T. Torres, M. Vogel, K. Barber and M. Valenstein (2011). "A randomized trial of telephonic counseling plus walking for depressed diabetes patients." Med Care **49**(7): 641-648.
Pill, R., N. C. Stott, S. R. Rollnick and M. Rees (1998). "A randomized controlled trial of an intervention designed to improve the care given in general practice to Type II diabetic patients: patient outcomes and professional ability to change behaviour." Fam Pract **15**(3): 229-235.
Polonsky, W. H., L. Fisher, C. H. Schikman, D. A. Hinnen, C. G. Parkin, Z. Jelsovsky, M. Axel-Schweitzer, B. Petersen and R. S. Wagner (2011). "A structured self-monitoring of blood glucose approach in type 2 diabetes encourages more frequent, intensive, and effective physician interventions: results from the STeP study." Diabetes Technol Ther **13**(8): 797-802.
Powers, B., J. M. Grubber, M. Olsen, M. Crowley and H. Bosworth (2012). "The cholesterol, hypertension, and glucose education (change) study: results from a randomized controlled trial in African Americans with diabetes." Journal of general internal medicine **27**: S316-S317.
Prestes, M., M. A. Gayarre, J. F. Elgart, L. Gonzalez, E. Rucci, J. M. Paganini and J. J. Gagliardino (2017). "Improving diabetes care at primary care level with a multistrategic approach: results of the DIAPREM programme." Acta Diabetol **54**(9): 853-861.
Ramallo-Fariña, Y., M. A. García-Bello, L. García-Pérez, M. Boronat, A. M. Wägner, L. Rodríguez-Rodríguez, P. de Pablos-Velasco, I. Llorente Gómez de Segura, H. González-Pacheco, M. Carmona Rodríguez and et al. (2020). "Effectiveness of Internet-Based Multicomponent Interventions for Patients and Health Care Professionals to Improve Clinical Outcomes in Type 2 Diabetes Evaluated Through the INDICA Study: multiarm Cluster Randomized Controlled Trial." JMIR mhealth and uhealth **8**(11): e18922.
Reutens, A. T., R. Hutchinson, T. Van Binh, C. Cockram, C. Deerochanawong, L. T. Ho, L. Ji, B. A. Khalid, A. P. Kong, M. A. Lim-Abrahan, C. E. Tan, A. Tjokroprawiro, K. H. Yoon, P. Z. Zimmet and J. E. Shaw (2012). "The

GIANT study, a cluster-randomised controlled trial of efficacy of education of doctors about type 2 diabetes mellitus management guidelines in primary care practice." Diabetes Res Clin Pract **98**(1): 38-45.
Ridgeway, N. A., D. R. Harvill, L. M. Harvill, T. M. Falin, G. M. Forester and O. D. Gose (1999). "Improved control of type 2 diabetes mellitus: a practical education/behavior modification program in a primary care clinic." South Med J **92**(7): 667-672.
Rubak, S., A. Sandbaek, T. Lauritzen, K. Borch-Johnsen and B. Christensen (2009). "General practitioners trained in motivational interviewing can positively affect the attitude to behaviour change in people with type 2 diabetes. One year follow-up of an RCT, ADDITION Denmark." Scand J Prim Health Care **27**(3): 172-179.
Sönnichsen, A. C., H. Winkler, M. Flamm, S. Panisch, P. Kowatsch, G. Klima, B. Fürthauer and R. Weitgasser (2010). "The effectiveness of the Austrian disease management programme for type 2 diabetes: a cluster-randomised controlled trial." BMC Fam Pract **11**: 86.
Sperl-Hillen, J. M., P. J. O'Connor, W. A. Rush, P. E. Johnson, T. Gilmer, G. Biltz, S. E. Asche and H. L. Ekstrom (2010). "Simulated physician learning program improves glucose control in adults with diabetes." Diabetes Care **33**(8): 1727-1733.
Stone, R. A., R. H. Rao, M. A. Sevick, C. Cheng, L. J. Hough, D. S. Macpherson, C. M. Franko, R. A. Anglin, D. S. Obrosky and F. R. Derubertis (2010). "Active care management supported by home telemonitoring in veterans with type 2 diabetes: the DiaTel randomized controlled trial." Diabetes Care **33**(3): 478-484.
Sturt, J. A., S. Whitlock, C. Fox, H. Hearnshaw, A. J. Farmer, M. Wakelin, S. Eldridge, F. Griffiths and J. Dale (2008). "Effects of the Diabetes Manual 1:1 structured education in primary care." Diabet Med **25**(6): 722-731.
Talavera, G. A., S. F. Castañeda, P. M. Mendoza, M. Lopez-Gurrola, S. Roesch, M. S. Pichardo, M. L. Garcia, F. Muñoz and L. C. Gallo (2021). "Latinos understanding the need for adherence in diabetes (LUNA-D): a randomized controlled trial of an integrated team-based care intervention among Latinos with diabetes." Transl Behav Med **11**(9): 1665-1675.
Tang, P. C., J. M. Overhage, A. S. Chan, N. L. Brown, B. Aghighi, M. P. Entwistle, S. L. Hui, S. M. Hyde, L. H. Klieman, C. J. Mitchell, A. J. Perkins, L. S. Qureshi, T. A. Waltimyer, L. J. Winters and C. Y. Young (2013). "Online disease management of diabetes: engaging and motivating patients online with enhanced resources-diabetes (EMPOWER-D), a randomized controlled trial." J Am Med Inform Assoc **20**(3): 526-534.
Taylor, K. I., K. M. Oberle, R. A. Crutcher and P. G. Norton (2005). "Promoting health in type 2 diabetes: nurse-physician collaboration in primary care." Biol Res Nurs **6**(3): 207-215.
Tourkmani, A. M., O. Abdelhay, H. I. Alkhashan, A. F. Alaboud, A. Bakhit, T. Elsaid, A. Alawad, A. Alobaikan, H. Alqahtani, A. Alqahtani, A. Mishriky, A. Bin Rsheed and T. J. Alharbi (2018). "Impact of an integrated care program on glycemic control and cardiovascular risk factors in patients with type 2 diabetes in Saudi Arabia: an interventional parallel-group controlled study." BMC Fam Pract **19**(1): 1.
van Bruggen, R., K. J. Gorter, R. P. Stolk, R. P. Verhoeven and G. E. H. M. Rutten (2008). "Implementation of locally adapted guidelines on type 2 diabetes." Family Practice **25**(6): 430-437.
van Dijk-de Vries, A., M. A. van Bokhoven, B. Winkens, B. Terluin, J. A. Knottnerus, T. van der Weijden and J. T. van Eijk (2015). "Lessons learnt from a cluster-randomised trial evaluating the effectiveness of Self-Management Support (SMS) delivered by practice nurses in routine diabetes care." BMJ Open **5**(6): e007014.
Varroud-Vial, M., D. Simon, J. Attali, I. Durand-Zaleski, L. Bera, C. Attali, C. Letondeur, K. Strauss, C. Petit and G. Charpentier (2004). "Improving glycaemic control of patients with Type 2 diabetes in a primary care setting: a French application of the Staged Diabetes Management programme." Diabet Med **21**(6): 592-598.
Wakefield, B. J., J. E. Holman, A. Ray, M. Scherubel, M. R. Adams, S. L. Hillis and G. E. Rosenthal (2011). "Effectiveness of home telehealth in comorbid diabetes and hypertension: a randomized, controlled trial." Telemed J E Health **17**(4): 254-261.
Weinberger, M., M. S. Kirkman, G. P. Samsa, E. A. Shortliffe, P. B. Landsman, P. A. Cowper, D. L. Simel and J. R. Feussner (1995). "A nurse-coordinated intervention for primary care patients with non-insulin-dependent diabetes mellitus: impact on glycemic control and health-related quality of life." J Gen Intern Med **10**(2): 59-66.

Weitzman, S., S. Greenfield, J. Billimek, T. Hava, P. Schvartzman, E. Yehiel, H. Tandeter, S. Eilat-Tsanani and S. H. Kaplan (2009). "Improving combined diabetes outcomes by adding a simple patient intervention to physician feedback: a cluster randomized trial." Isr Med Assoc J **11**(12): 719-724.

Welch, G., N. A. Allen, S. E. Zagarins, K. D. Stamp, S. E. Bursell and R. J. Kedziora (2011). "Comprehensive diabetes management program for poorly controlled Hispanic type 2 patients at a community health center." Diabetes Educ **37**(5): 680-688.

Welch, G., S. E. Zagarins, P. Santiago-Kelly, Z. Rodriguez, S. E. Bursell, M. C. Rosal and R. A. Gabbay (2015). "An internet-based diabetes management platform improves team care and outcomes in an urban Latino population." Diabetes Care **38**(4): 561-567.

Whitehead, L. C., M. T. Crowe, J. D. Carter, V. R. Maskill, D. Carlyle, C. Bugge and C. M. A. Frampton (2017). "A nurse-led education and cognitive behaviour therapy-based intervention among adults with uncontrolled type 2 diabetes: a randomised controlled trial." Journal of evaluation in clinical practice **23**(4): 821-829.

Wild, S. H., J. Hanley, S. C. Lewis, J. A. McKnight, L. B. McCloughan, P. L. Padfield, R. A. Parker, M. Paterson, H. Pinnock, A. Sheikh and et al. (2016). "Supported Telemonitoring and Glycemic Control in People with Type 2 Diabetes: the Telescot Diabetes Pragmatic Multicenter Randomized Controlled Trial." PLoS medicine **13**(7): e1002098.

Wollny, A., A. Altiner, A. Daubmann, E. Drewelow, C. Helbig, S. Löscher, M. Pentzek, S. Santos, K. Wegscheider, S. Wilm and C. Löffler (2019). "Patient-centered communication and shared decision making to reduce HbA1c levels of patients with poorly controlled type 2 diabetes mellitus - results of the cluster-randomized controlled DEBATE trial." BMC Fam Pract **20**(1): 87.

Woodard, L., A. B. Amspoker, N. E. Hundt, H. S. Gordon, B. Hertz, E. Odom, A. Utech, J. Razjouyan, S. S. Rajan, N. Kamdar, J. Lindo, L. Kiefer, P. Mehta and A. D. Naik (2022). "Comparison of Collaborative Goal Setting With Enhanced Education for Managing Diabetes-Associated Distress and Hemoglobin A1c Levels: A Randomized Clinical Trial." JAMA Netw Open **5**(5): e229975.

Xu, C., Z. Dong, P. Zhang, G. Chang, Q. Xiang, M. Zhang, J. Zhou, C. Qiao, Q. Yang, Y. Qin and P. Lou (2021). "Effect of group cognitive behavioural therapy on psychological stress and blood glucose in people with type 2 diabetes mellitus: A community-based cluster randomized controlled trial in China." Diabetic Medicine **38**(2): 1-15.

Yang, Y., E. Y. Lee, H. S. Kim, S. H. Lee, K. H. Yoon and J. H. Cho (2020). "Effect of a Mobile Phone-Based Glucose-Monitoring and Feedback System for Type 2 Diabetes Management in Multiple Primary Care Clinic Settings: Cluster Randomized Controlled Trial." JMIR Mhealth Uhealth **8**(2): e16266.

Young, H. M., S. Miyamoto, M. Dharmar and Y. Tang-Feldman (2020). "Nurse Coaching and Mobile Health Compared With Usual Care to Improve Diabetes Self-Efficacy for Persons With Type 2 Diabetes: Randomized Controlled Trial." JMIR Mhealth Uhealth **8**(3): e16665.

Young, R. J., J. Taylor, T. Friede, S. Hollis, J. M. Mason, P. Lee, E. Burns, A. F. Long, T. Gambling, J. P. New and J. M. Gibson (2005). "Pro-active call center treatment support (PACCTS) to improve glucose control in type 2 diabetes: a randomized controlled trial." Diabetes Care **28**(2): 278-282.

Zandiyeh, Z., B. Hedayati and E. Zare (2015). "Effect of public health nurses' educational intervention on self-care of the patients with type 2 diabetes." J Educ Health Promot **4**: 88.

Zhang, H. Z., P. Zhang, G. Q. Chang, Q. Y. Xiang, H. Cao, J. Y. Zhou, Z. M. Dong, C. Qiao, C. R. Xu, Y. Qin and P. A. Lou (2021). "Effectiveness of cognitive behavior therapy for sleep disturbance and glycemic control in persons with type 2 diabetes mellitus: A community-based randomized controlled trial in China." World J Diabetes **12**(3): 292-305.
